# Effect of semaglutide on arrhythmic, major cardiovascular, and renal outcomes in patients with overweight or obesity: a systematic review and meta-analysis

**DOI:** 10.1186/s40001-025-03124-y

**Published:** 2025-09-02

**Authors:** Rui Wu, Bo Xing, Zijun Zhou, Liming Yu, Huishan Wang

**Affiliations:** 1 School of Life Sciences and Biopharmaceuticals, Shenyang Pharmaceutical University, 103 Wenhua Road, Shenyang, 110016 Liaoning People’s Republic of China; 2 State Key Laboratory of Frigid Zone Cardiovascular Disease, Department of Cardiovascular Surgery, General Hospital of Northern Theater Command, 83 Wenhua Road, Shenyang, 110016 Liaoning People’s Republic of China

**Keywords:** Semaglutide, Arrhythmic, Cardiovascular, Renal, Overweight or obesity, Meta-analysis

## Abstract

**Background:**

Semaglutide has demonstrated potential in controlling hyperglycemia and lowering cardiovascular (CV) risk. However, its impact on arrhythmic, major CV, and renal outcomes is not well-defined. This systematic review and meta-analysis aimed to assess these effects in patients with overweight or obesity.

**Methods:**

We searched the PubMed, Embase, and Cochrane databases for eligible randomized controlled trials (RCTs) reported up to January 2025. We calculated overall relative risks (RRs) with 95% confidence intervals (CIs) for these outcomes. In addition, subgroup analyses were performed based on age, treatment duration and obesity level.

**Results:**

Ten RCTs involving 22,937 patients were included. Compared with the controls, semaglutide significantly reduced the risk of atrial fibrillation (AF) (RR 0.79, 95% CI 0.63–0.99), sinus node dysfunction (RR 0.43, 95% CI 0.19–1.00), acute myocardial infarction (RR 0.72, 95% CI 0.60–0.85), and angina pectoris (RR 0.77, 95% CI 0.61–0.98). Subgroup analyses revealed greater efficacy in patients over 60 years old and those treated for more than 52 weeks, especially for acute myocardial infarction, angina pectoris, and acute kidney injury.

**Conclusion:**

Semaglutide reduces the risk of AF, sinus node dysfunction, acute myocardial infarction, and angina pectoris in patients with overweight or obesity. However, its effects on other arrhythmic, CV, and renal outcomes remain uncertain.

**Supplementary Information:**

The online version contains supplementary material available at 10.1186/s40001-025-03124-y.

## Introduction

Obesity and overweight are global health challenges associated with increased risks of cardiovascular (CV) diseases, arrhythmias, and renal complications [1, 2]. These conditions significantly contribute to morbidity and mortality worldwide; obesity is not only a risk factor for coronary artery disease, congestive heart failure, and stroke but also influences heart rhythm disturbances [3–5]. This underscores the urgent need for effective therapeutic interventions that can address both weight management and associated CV outcomes.

Semaglutide, a glucagon-like peptide-1 (GLP-1) receptor agonist, has emerged as a promising treatment for weight management and metabolic control [6]. GLP-1 receptor agonists improve glucose metabolism, enhance insulin secretion, and promote satiety, which contribute to weight loss. Furthermore, these agents exert CV benefits by improving endothelial function, reducing inflammation, and lowering blood pressure—mechanisms that are particularly relevant in the context of obesity-related CV disease [7]. Given these multifaceted benefits, semaglutide presents a compelling candidate for addressing the complex health needs of patients with obesity or overweight.

Recent randomized controlled trials (RCTs) have demonstrated that semaglutide not only promotes weight loss but also reduces the risk of major adverse CV outcomes in patients with type 2 diabetes [8, 9]. These findings have sparked interest in its potential applications for patients with obesity or overweight, particularly those at high risk for CV and renal complications. However, the evidence regarding its effects on specific outcomes, such as atrial fibrillation (AF), acute myocardial infarction, and chronic kidney disease, is still limited and inconsistent. Given the increasing prevalence of obesity and its associated comorbidities, a comprehensive evaluation of the impact of semaglutide on these outcomes is crucial for guiding clinical practice.

In this context, our meta-analysis aims to synthesize the available evidence from RCTs to assess the effects of subcutaneous semaglutide on arrhythmic, major CV, and renal outcomes in patients with overweight or obesity. We chose to focus on semaglutide among the GLP-1 receptor agonists due to its robust efficacy and favorable safety profile observed in clinical trials, positioning it as a leading option for both weight management and CV risk reduction. By focusing on this population, we sought to clarify whether semaglutide offers protective benefits beyond weight reduction, particularly in reducing the risk of arrhythmias, CV events, and renal complications. In addition, we explored potential subgroup differences based on age, treatment duration and obesity level to identify populations that may derive the greatest benefit from semaglutide therapy.

The findings of this study have important implications for the management of obesity and its associated complications. By providing a comprehensive assessment of the effects of semaglutide on a range of clinical outcomes, this meta-analysis aims to inform healthcare providers and policymakers about the potential role of semaglutide in improving the health of patients with overweight or obesity. Furthermore, it highlights areas where further research is needed to optimize the use of semaglutide in this population.

## Methods

This meta-analysis was performed according to the Preferred Reporting Items for Systematic Reviews and Meta-Analyses (PRISMA) guidelines [10] and has been registered at PROSPERO (CRD42025638461).

### Data sources and search strategy

A thorough search was performed in the PubMed, Embase, and the Cochrane Library until 20 January 2025. The keywords searched were overweight or obesity, semaglutide, and RCTs. The search strategy can be found in the Supplementary Table 1. No language restrictions were applied; studies published in any language were considered for inclusion. Disagreements between reviewers (RW and BX) during the search process regarding the eligibility of studies were resolved through discussion, and a third reviewer (ZJZ) was consulted when necessary to ensure consensus.

### Inclusion and exclusion criteria

The inclusion criteria were as follows: (1) RCTs with an interventional design; (2) patients diagnosed with overweight or obesity; (3) reporting outcomes related to arrhythmic, major CV, or renal outcomes; and (4) comparison of semaglutide in the intervention group versus placebo or other medications in the control group. Studies were excluded if they involved unpublished data, duplicate publications, case reports, or conference abstracts.

### Quality assessment

The Cochrane Collaboration’s risk of bias tool [11] was employed to evaluate bias in individual trials. This tool identifies potential bias across seven domains: selection bias, performance bias, detection bias, attrition bias, reporting bias, and other biases. Each study was classified as having a high, unclear, or low risk of bias in these areas. The quality assessment was conducted by two independent reviewers (RW and BX) to ensure reliability. Discrepancies were resolved through discussion, and a third reviewer (ZJZ) was involved when necessary.

### Data extraction

From the included studies, key data were extracted, including the first author, publication year, NCT number, patient demographics (age, weight, BMI, waist circumference), sample size, treatment comparisons, and treatment durations. Discrepancies during the data extraction process were discussed between the reviewers (RW and BX), and if necessary, a third reviewer (ZJZ) was consulted to reach a consensus. The analysis focused on three primary outcome categories: arrhythmic outcomes, major CV outcomes, and renal outcomes. The arrhythmic outcomes included AF, atrial flutter (AFL), atrial tachycardia, bradyarrhythmia, sinus node dysfunction, supraventricular tachycardia, ventricular tachycardia, sinus bradycardia, and cardiac arrest; major CV outcomes included acute myocardial infarction, angina unstable, coronary artery disease, angina pectoris, cardiac failure congestive, myocardial infarction, aortic valve stenosis, cardiac failure, cardiac failure acute, myocarditis, and palpitations; and renal outcomes included nephrolithiasis, acute kidney injury, ureterolithiasis, chronic kidney disease, calculus urinary, hydronephrosis, renal colic, and renal failure.

### Statistical analysis

Dichotomous outcomes are quantified by calculating relative risks (RRs) coupled with 95% confidence intervals (CIs). All included studies reported RR as the primary effect measure. Therefore, we uniformly used RR for analysis, and there were no other effect measures (such as hazard ratios or odds ratios) that needed to be harmonized. To account for variability among study populations and outcomes, a random-effects model was applied to pool effect estimates [12]. A random-effects model is appropriate in meta-analyses when heterogeneity is expected due to differences in study design, populations, and interventions. This model allows for a more generalized effect size, reflecting the variability across the studies and providing a more comprehensive overview of the treatment effect.

Heterogeneity was evaluated via the chi-square test and *I*^2^ statistic. For the subgroup analyses, actual *I*^2^ values were calculated and are reported for each outcome to allow to independently assess consistency. Subgroup analyses were performed to explore the effects of age, treatment duration and obesity level on outcomes. The rationale for conducting subgroup analyses stems from the recognition that age and treatment duration are critical factors influencing treatment efficacy. Older patients may have different responses to interventions due to the presence of comorbidities, which can affect CV risk profiles. Furthermore, extending the duration of treatment may lead to enhanced therapeutic effects, as the benefits of semaglutide may accumulate over time. In addition, we further categorized patients based on obesity levels defined by body mass index (BMI): Class I (BMI 30–34.9 kg/m^2^), Class II (BMI 35–39.9 kg/m^2^), and Class III (BMI ≥ 40 kg/m^2^). This classification allows for a more nuanced understanding of how the efficacy of semaglutide may vary among patients with different degrees of obesity. Sensitivity analyses were conducted to evaluate the robustness of our findings. Specifically, we identified studies that had a substantial influence on overall effect sizes by conducting a leave-one-out analysis. In this approach, each study was sequentially removed from the analysis to determine its impact on the pooled results for key outcomes. This process allowed us to assess whether our findings were consistent and if any single study disproportionately affected the overall conclusions. Publication bias was assessed via Egger’s test, which evaluates the asymmetry of the funnel plot for the primary outcomes. Statistical significance was set at *p* values < 0.05. All analyses were performed via Stata software version 12.0 (StataCorp, College Station, TX, USA).

## Results

### Study selection and characteristics

**Fig. 1:**
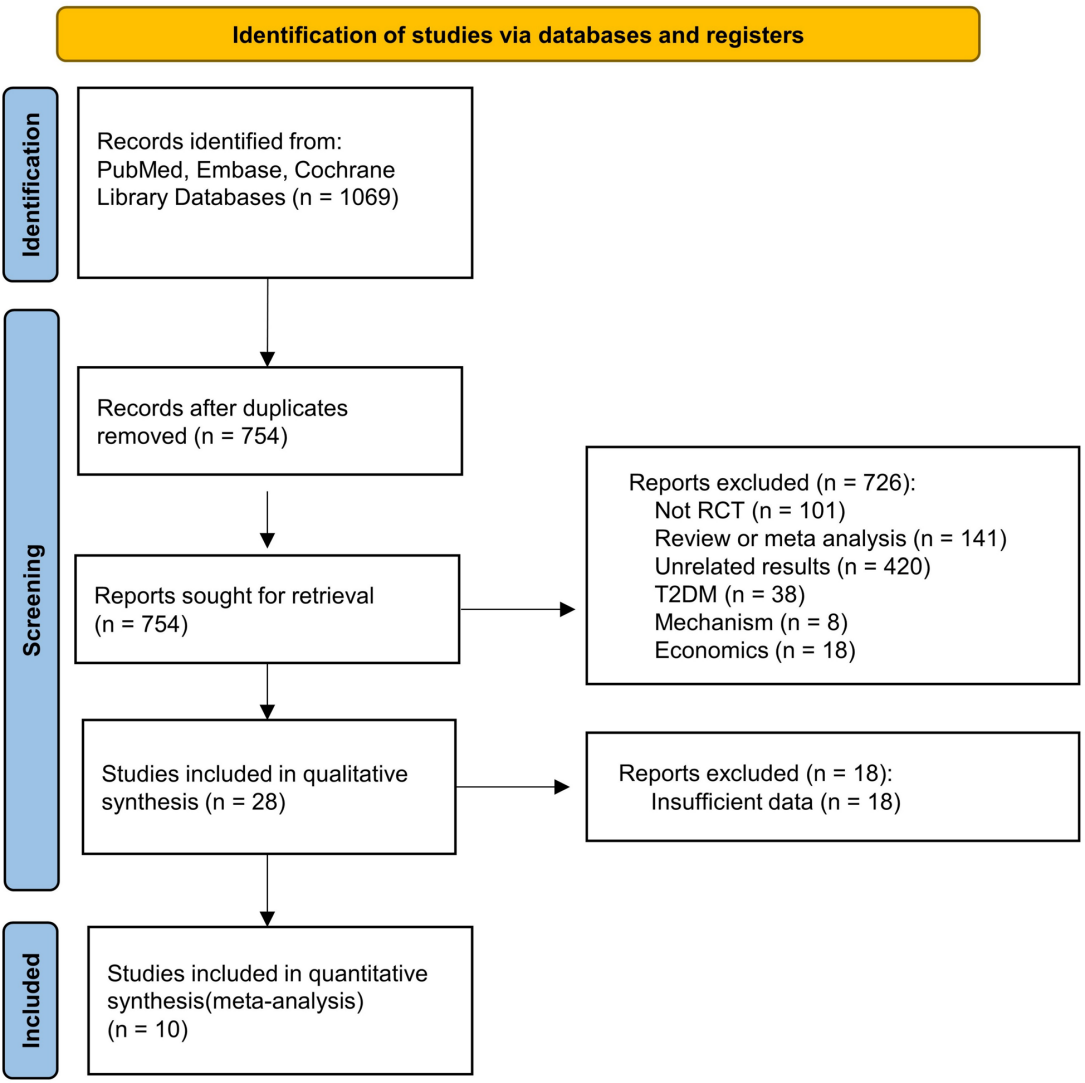
Flowchart of study selection

The initial database search yielded 1069 articles. After removing duplicates, 754 studies were retained for screening. Of these, 726 were excluded for being irrelevant reviews, non-RCTs, or other reasons. Following a full-text review of the remaining 28 articles, 18 were excluded due to insufficient data. Ultimately, 10 studies were included in the meta-analysis [13–22]. The study selection process is illustrated in Fig. 1, and the baseline characteristics of the included studies are summarized in Table 1.

**Table 1:** Basic characteristics of the eligible studies

First author	Year	Acronym	NCT number	Age (yr)	Weight (kg)	BMI (kg/m^2^)	Waist circumference (cm)	Semaglutide intervention	Semaglutide dose (mg)	Frequency	Control intervention	Semaglutide group (N)	Controlgroup (N)	Duration (w)	Reference
Wilding	2021	STEP 1	NCT03548935	46.3	105.3	37.9	114.7	Subcutaneous	2.4	Once-weekly	Placebo	1306	655	68	[15]
Wadden	2021	STEP 3	NCT03611582	46	105.8	38	113	Subcutaneous	2.4	Once-weekly	Placebo	407	204	68	[14]
Rubino	2021	STEP 4	NCT03548987	46.7	96.1	34.4	105.2	Subcutaneous	2.4	Once-weekly	Placebo	535	268	68	[13]
Garvey	2022	STEP 5	NCT03693430	47.4	106.1	38.6	115.8	Subcutaneous	2.4	Once-weekly	Placebo	152	152	104	[16]
Rubino	2022	STEP 8	NCT04074161	49.2	105	37.7	113.3	Subcutaneous	2.4	Once-weekly	Placebo	126	85	68	[18]
Kadowaki	2022	STEP 6	NCT03811574	51.3	88	32	103.8	Subcutaneous	2.4	Once-weekly	Placebo	199	101	68	[17]
Kosiborod	2023	STEP-HFpEF	NCT04788511	69	105.1	37	119.4	Subcutaneous	2.4	Once-weekly	Placebo	263	266	52	[19]
Lincoff	2023	SELECT	NCT03574597	61.6	96.6	33.3	111.3	Subcutaneous	2.4	Once-weekly	Placebo	8803	8801	208	[20]
Bliddal	2024	STEP 9	NCT05064735	56	108.6	40.3	118.7	Subcutaneous	2.4	Once-weekly	Placebo	271	136	68	[21]
McGowan	2024	STEP 10	NCT05040971	53	111.6	40.1	120.1	Subcutaneous	2.4	Once-weekly	Placebo	138	69	52	[22]

The included studies involved the subcutaneous administration of semaglutide at doses of up to 2.4 mg. The participants’ ages ranged from 46 to 69 years, with treatment durations spanning 52–208 weeks. A total of 22,937 patients with overweight or obesity were enrolled, with 12,200 in the semaglutide group and 10,737 in the control group.

### Arrhythmic outcomes

**Fig. 2:**
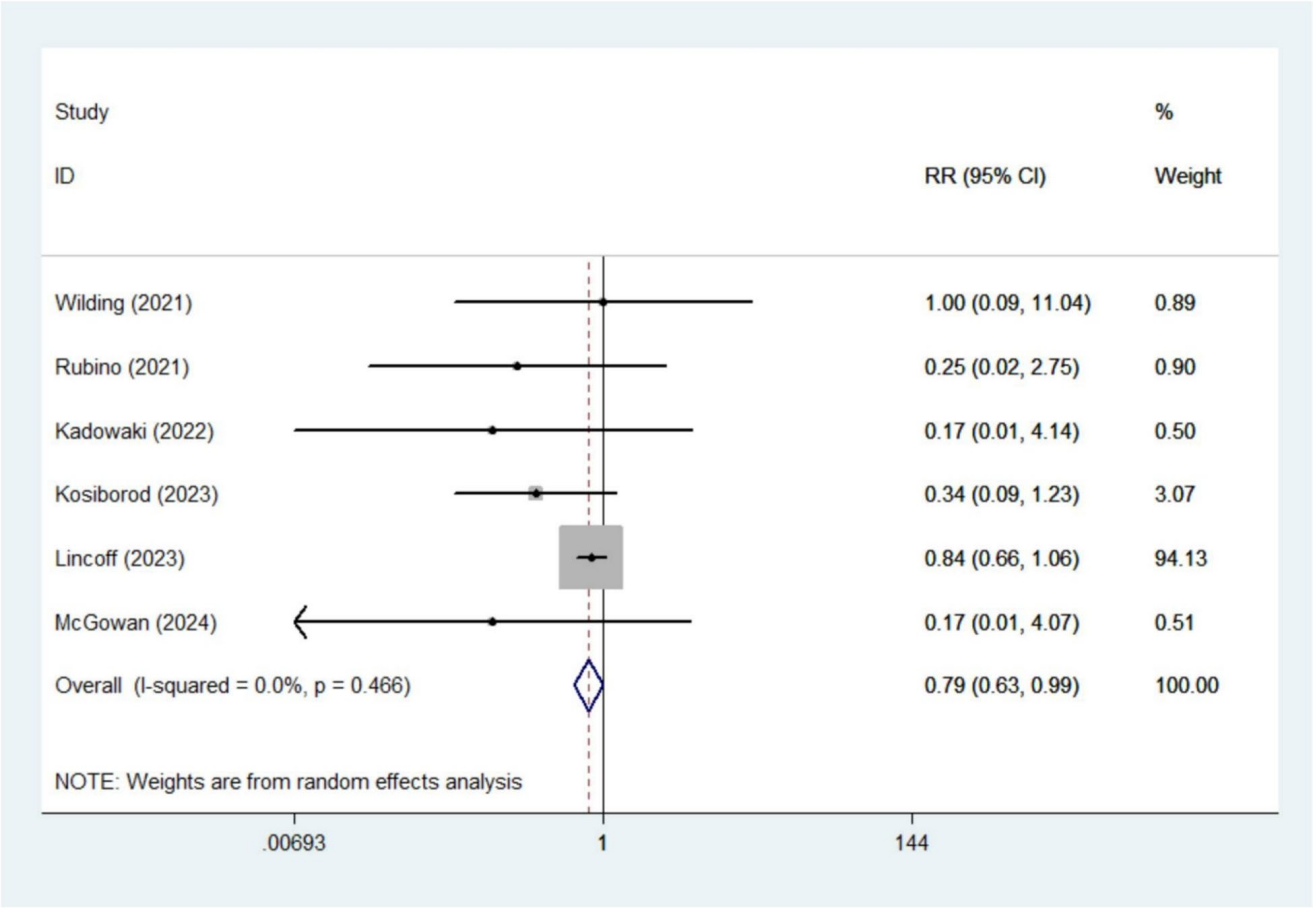
Forest plot of atrial fibrillation

**Fig. 3:**
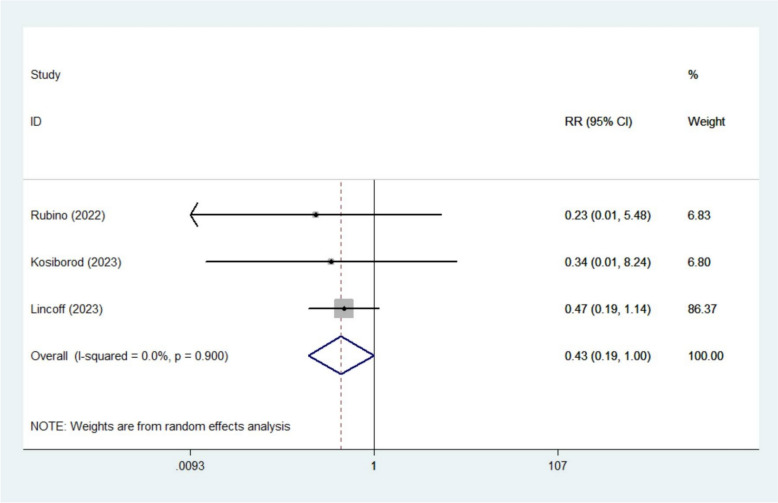
Forest plot of sinus node dysfunction

The meta-analysis revealed that semaglutide significantly lowered the risk of AF (RR 0. 79, 95% CI 0.63–0.99; *I*^2^ 0%, 6 studies [13, 15, 17, 19, 20, 22]) (Fig. 2) and sinus node dysfunction (RR 0.43, 95% CI 0.19–1.00; *I*^2^ 0%, 3 studies [18–20]) (Fig. 3). However, no significant benefits were observed for other arrhythmic outcomes, including AFL (RR 0.65, 95% CI 0.25–1.71; *I*^2^ 15.5%, 4 studies [18–20, 22]), atrial tachycardia (RR 0.38, 95% CI 0.10–1.41; *I*^2^ 0%, 3 studies [15, 20, 22]), bradyarrhythmia (RR 0.83, 95% CI 0.35–1.95; *I*^2^ 0%, 3 studies [18–20]), supraventricular tachycardia (RR 2.18, 95% CI 0.96–4.91; *I*^2^ 0%, 3 studies [13, 19, 20]), ventricular tachycardia (RR 0.85, 95% CI 0.49–1.49; *I*^2^ 2.4%, 3 studies [19, 20, 22]), sinus bradycardia (RR 0.80, 95% CI 0.22–2.99; *I*^2^ 0%, 2 studies [19, 20]) or cardiac arrest (RR 0.77, 95% CI 0.46–1.28; *I*^2^ 0%, 2 studies [19, 20]) (Figures S1–S7).

### Major CV outcomes

**Fig. 4:**
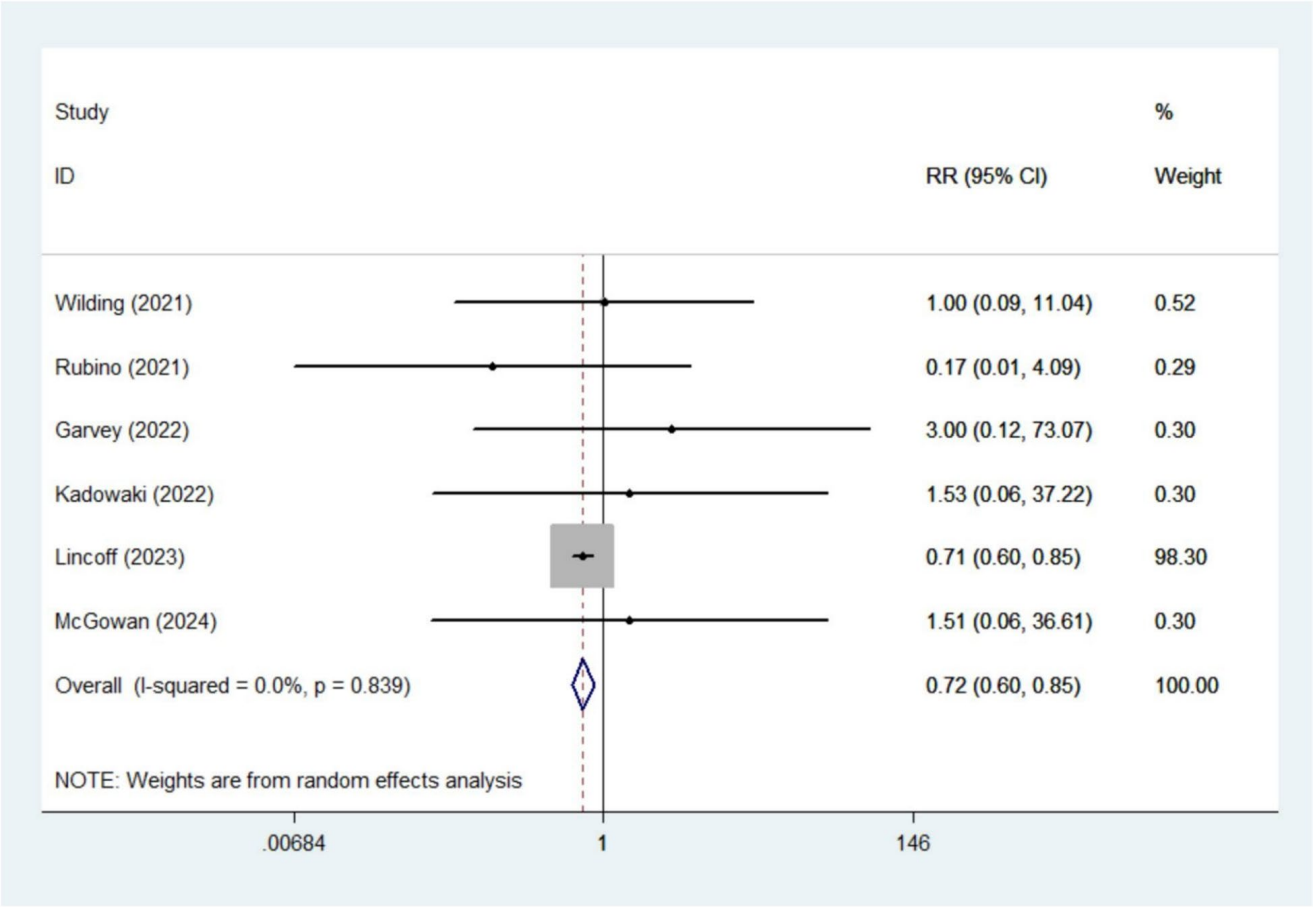
Forest plot of acute myocardial infarction

**Fig. 5:**
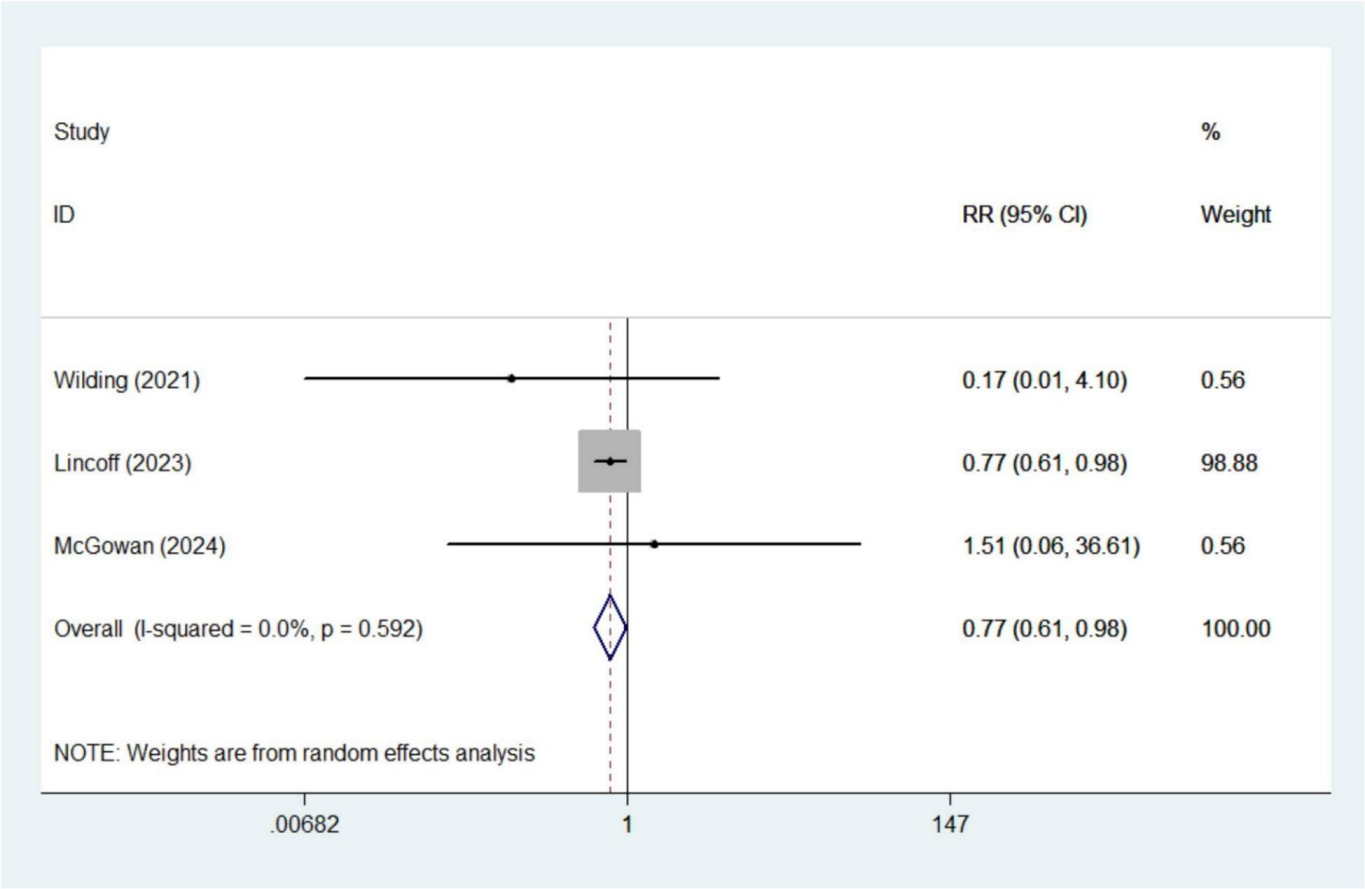
Forest plot of angina pectoris

The meta-analysis demonstrated that semaglutide significantly reduced the risk of acute myocardial infarction (RR 0.72, 95% CI 0.60–0.85; *I*^2^ 0%, 6 studies [13, 15–17, 20, 22]) (Fig. 4) and angina pectoris (RR 0.77, 95% CI 0.61–0.98; *I*^2^ 0%, 3 studies [15, 20, 22]) (Fig. 5). However, no significant effects were observed for other CV outcomes, including angina unstable (RR 0.87, 95% CI 0.72–1.06; *I*^2^ 0%, 4 studies [15, 19–21]), coronary artery disease (RR 0.76, 95% CI 0.57–1.01; *I*^2^ 0%, 4 studies [15, 18–20]), cardiac failure congestive (RR 0.80, 95% CI 0.50–1.28; *I*^2^ 0.1%, 3 studies [18–20]), myocardial infarction (RR 0.54, 95% CI 0.06–4.84; *I*^2^ 58.3%, 2 studies [15, 20]), aortic valve stenosis (RR 1.29, 95% CI 0.46–3.62; *I*^2^ 0%, 2 studies [19, 20]), cardiac failure (RR 0.25, 95% CI 0.01–4.52; *I*^2^ 77.4%, 2 studies [19, 20]), cardiac failure acute (RR 0.75, 95% CI 0.48–1.17; *I*^2^ 0%, 2 studies [19, 20]), myocarditis (RR 0.24, 95% CI 0.02–2.27; *I*^2^ 0%, 2 studies [15, 20]), or palpitations (RR 0.41, 95% CI 0.12–1.39; *I*^2^ 0%, 2 studies [15, 20]) (Figures S8–S16).

### Renal outcomes

**Fig. 6:**
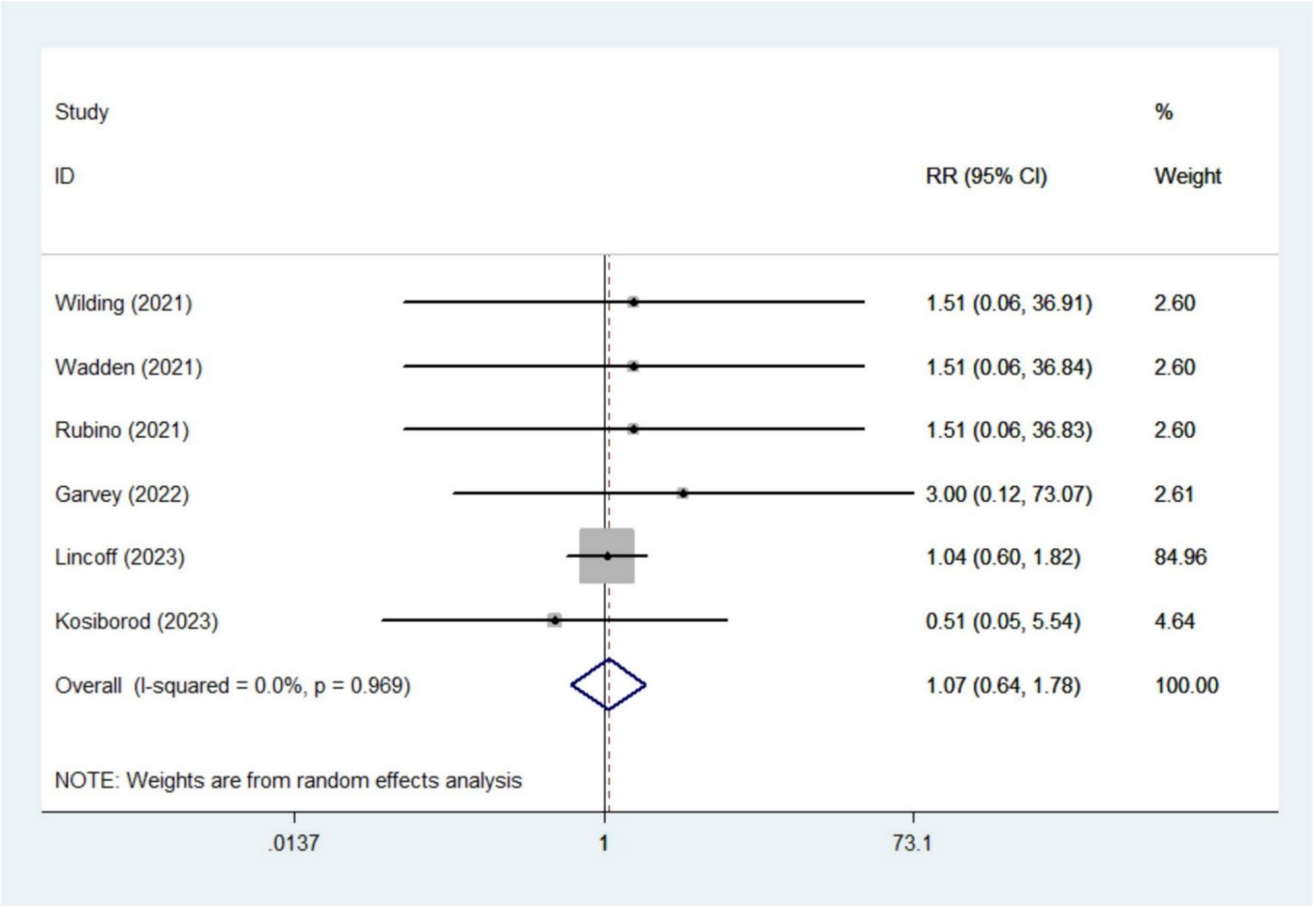
Forest plot of nephrolithiasis

**Fig. 7:**
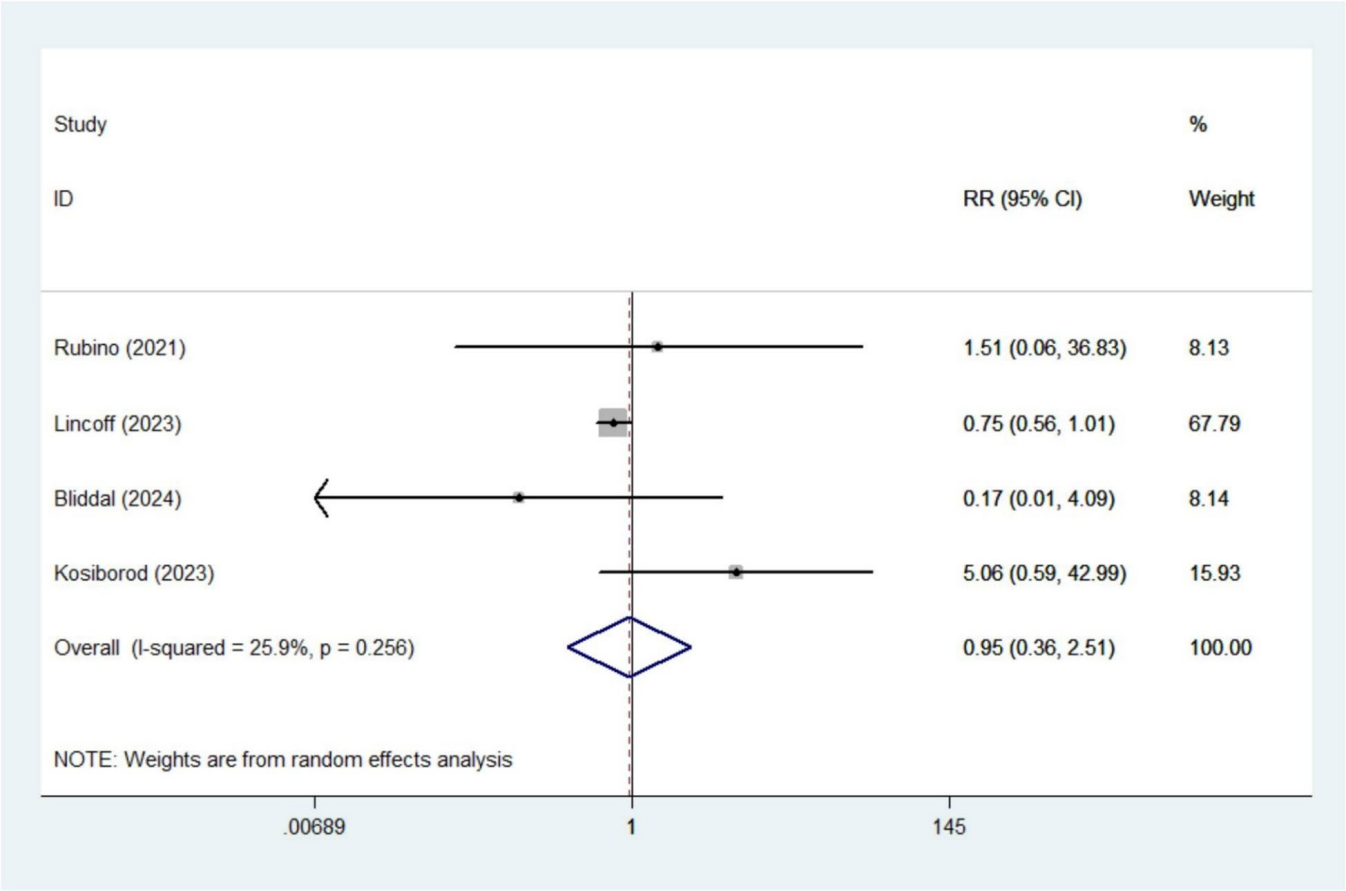
Forest plot of acute kidney injury

The meta-analysis revealed no significant impact of semaglutide on renal outcomes, including nephrolithiasis (RR 1.07, 95% CI 0.64–1.78; *I*^2^ 0%, 6 studies [13–16, 19, 20]) (Fig. 6), acute kidney injury (RR 0.95, 95% CI 0.36–2.51; *I*^2^ 25.9%, 4 studies [13, 19–21]) (Fig. 7), ureterolithiasis (RR 1.80, 95% CI 0.88–3.67; *I*^2^ 0%, 3 studies [13, 17, 20]), chronic kidney disease (RR 0.90, 95% CI 0.33–2.43; *I*^2^ 0%, 2 studies [19, 20]), calculus urinary (RR 1.72, 95% CI 0.55–5.40; *I*^2^ 0%, 2 studies [15, 20]), hydronephrosis (RR 1.91, 95% CI 0.54–6.82; *I*^2^ 0%, 2 studies [14, 20]), renal colic (RR 1.70, 95% CI 0.13–22.66; *I*^2^ 46.9%, 2 studies [19, 20]), or renal failure (RR 1.81, 95% CI 0.39–8.35; *I*^2^ 35.4%, 2 studies [19, 20]) (Figures S17–S22).

### Subgroup analysis

Subgroup analyses were conducted based on patient age, specifically comparing those aged over 60 years to younger patients. We determined the age subgroup based on the explicit inclusion criteria of each trial that reported age-distribution data. Notably, all studies included individuals aged 60 years and older, thus providing a consistent basis for this subgroup analysis. Treatment duration was analyzed with similar parameters. This approach limits potential confounding bias, allowing us to evaluate the impact of age and treatment duration more precisely on the effectiveness of semaglutide (Table 2). The results indicated that semaglutide administration exceeding 52 weeks led to a notable reduction in the risk of acute myocardial infarction (RR 0.71, 95% CI 0.60–0.85; *I*^2^ 0%), angina pectoris (RR 0.76, 95% CI 0.60–0.97; *I*^2^ 0%), and acute kidney injury (RR 0.74, 95% CI 0.56–0.99; *I*^2^ 0%). Furthermore, in patients aged over 60 years, semaglutide significantly lowered the risk of acute myocardial infarction (RR 0.71, 95% CI 0.59–0.85), coronary artery disease (RR 0.74, 95% CI 0.56–0.99; *I*^2^ 0%), and angina pectoris (RR 0.77, 95% CI 0.60–0.98). For other outcomes, no significant risk reduction was observed based on age or treatment duration. Overall, semaglutide appeared to demonstrate enhanced efficacy in patients over 60 years of age and those with longer treatment durations. Heterogeneity was negligible in most subgroups, suggesting strong consistency, though caution is warranted due to the limited number of studies included. In addition, in our subgroup analysis based on obesity levels defined by BMI, semaglutide demonstrated significant efficacy primarily in patients with Class I obesity (BMI 30–34.9 kg/m^2^), where it was associated with a notable reduction in the risk of acute myocardial infarction (RR 0.72, 95% CI 0.60–0.86; *I*^2^ 0%), coronary artery disease (RR 0.75, 95% CI 0.56–0.99), and angina pectoris (RR 0.78, 95% CI 0.61–0.99). Conversely, for patients with Class II obesity (BMI 35–39.9 kg/m^2^) and Class III obesity (BMI ≥ 40 kg/m^2^), the results showed limited efficacy and a lack of clear evidence of benefit. Overall, these findings suggest that semaglutide has some efficacy for patients with Class I obesity.

**Table 2:** Subgroup analyses based on age, treatment duration and obesity level

Subgroup	No. of studies	RR (95% CI)	Heterogeneity (*I*^2^%)	*p*
Atrial fibrillation				
Age ≤ 60 yr	4	0.33 (0.09, 1.18)	0	0.75
Age > 60 yr	2	0.80 (0.64, 1.01)	45.3	0.176
Duration ≤ 52 w	2	0.30 (0.09, 1.00)	0	0.691
Duration > 52 w	4	0.81 (0.65, 1.03)	0	0.588
Class I (BMI 30–34.9 kg/m^2^)	3	0.82 (0.65, 1.04)	0	0.097
Class II (BMI 35–39.9 kg/m^2^)	2	0.44 (0.14, 1.37)	0	0.157
Class III (BMI ≥ 40 kg/m^2^)	1	0.17 (0.01, 4.13)	Not estimable	0.276
Atrial flutter				
Age ≤ 60 yr	2	0.19 (0.02, 1.85)	0	0.898
Age > 60 yr	2	0.92 (0.52, 1.61)	40.7	0.194
Duration ≤ 52 w	2	0.15 (0.01, 1.37)	0	0.945
Duration > 52 w	2	0.98 (0.55, 1.73)	0	0.354
Class I (BMI 30–34.9 kg/m^2^)	1	1.05 (0.58, 1.87)	Not estimable	0.882
Class II (BMI 35–39.9 kg/m^2^)	2	0.18 (0.02, 1.57)	0	0.121
Class III (BMI ≥ 40 kg/m^2^)	1	0.17 (0.01, 4.13)	Not estimable	0.276
Atrial tachycardia				
Age ≤ 60 yr	2	0.50 (0.07, 3.55)	0	0.341
Age > 60 yr	1	0.33 (0.06, 1.65)	Not estimable	Not estimable
Duration ≤ 52 w	1	0.16 (0.01, 4.06)	Not estimable	Not estimable
Duration > 52 w	2	0.45 (0.11, 1.77)	0	0.409
Class I (BMI 30–34.9 kg/m^2^)	1	0.33 (0.07, 1.65)	Not estimable	0.178
Class II (BMI 35–39.9 kg/m^2^)	1	1.51 (0.06, 36.88)	Not estimable	0.802
Class III (BMI ≥ 40 kg/m^2^)	1	0.17 (0.01, 4.13)	Not estimable	0.276
Bradyarrhythmia				
Age ≤ 60 yr	1	0.22 (0.01, 5.47)	Not estimable	Not estimable
Age > 60 yr	2	0.90 (0.37, 2.17)	0	0.521
Duration ≤ 52 w	1	0.33 (0.01, 8.23)	Not estimable	Not estimable
Duration > 52 w	2	0.87 (0.36, 2.07)	0	0.379
Class I (BMI 30–34.9 kg/m^2^)	1	1.00 (0.40, 2.52)	Not estimable	1
Class II (BMI 35–39.9 kg/m^2^)	2	0.28 (0.03, 2.66)	0	0.266
Sinus node dysfunction				
Age ≤ 60 yr	1	0.22 (0.01, 5.47)	Not estimable	Not estimable
Age > 60 yr	2	0.45 (0.19, 1.07)	0	0.848
Duration ≤ 52 w	1	0.33 (0.01, 8.23)	Not estimable	Not estimable
Duration > 52 w	2	0.44 (0.18, 1.04)	0	0.667
Class I (BMI 30–34.9 kg/m^2^)	1	0.47 (0.19, 1.15)	Not estimable	0.096
Class II (BMI 35–39.9 kg/m^2^)	2	0.28 (0.03, 2.65)	0	0.266
Supraventricular tachycardia				
Age ≤ 60 yr	1	1.50 (0.06, 36.83)	Not estimable	Not estimable
Age > 60 yr	2	2.17 (0.96, 4.90)	30.9	0.229
Duration ≤ 52 w	1	0.33 (0.01, 8.23)	Not estimable	Not estimable
Duration > 52 w	2	2.47 (1.07, 5.74)	0	0.752
Class I (BMI 30–34.9 kg/m^2^)	2	2.47 (1.07, 5.74)	0	0.035
Class II (BMI 35–39.9 kg/m^2^)	1	0.34 (0.01, 8.27)	Not estimable	0.506
Ventricular tachycardia				
Age ≤ 60 yr	1	0.16 (0.01, 4.06)	Not estimable	Not estimable
Age > 60 yr	2	0.90 (0.57, 1.40)	0	0.322
Duration ≤ 52 w	2	0.18 (0.02, 1.69)	0	0.933
Duration > 52 w	1	0.94 (0.60, 1.49)	Not estimable	Not estimable
Class I (BMI 30–34.9 kg/m^2^)	1	0.95 (0.60, 1.49)	Not estimable	0.816
Class II (BMI 35–39.9 kg/m^2^)	1	0.20 (0.01, 4.23)	Not estimable	0.304
Class III (BMI ≥ 40 kg/m^2^)	1	0.17 (0.01, 4.13)	Not estimable	0.276
Acute myocardial infarction				
Age ≤ 60 yr	5	1.00 (0.30, 3.33)	0	0.775
Age > 60 yr	1	0.71 (0.59, 0.85)	Not estimable	Not estimable
Duration ≤ 52 w	1	1.51 (0.06, 36.61)	Not estimable	Not estimable
Duration > 52 w	5	0.71 (0.60, 0.85)	0	0.761
Class I (BMI 30–34.9 kg/m^2^)	3	0.72 (0.60, 0.86)	0	0
Class II (BMI 35–39.9 kg/m^2^)	2	1.49 (0.22, 10.11)	0	0.686
Class III (BMI ≥ 40 kg/m^2^)	1	1.50 (0.06, 36.35)	Not estimable	0.803
Angina unstable				
Age ≤ 60 yr	2	1.50 (0.15, 14.46)	0	0.999
Age > 60 yr	2	0.86 (0.71, 1.05)	0	0.561
Duration ≤ 52 w	1	0.33 (0.01, 8.23)	Not estimable	Not estimable
Duration > 52 w	3	0.87 (0.71, 1.06)	0	0.893
Class I (BMI 30–34.9 kg/m^2^)	1	0.87 (0.72, 1.06)	Not estimable	0.176
Class II (BMI 35–39.9 kg/m^2^)	2	0.71 (0.07, 6.84)	0	0.769
Class III (BMI ≥ 40 kg/m^2^)	1	1.51 (0.06, 36.71)	Not estimable	0.802
Coronary artery disease				
Age ≤ 60 yr	2	1.75 (0.18, 16.68)	0	0.897
Age > 60 yr	2	0.74 (0.56, 0.99)	0	0.83
Duration ≤ 52 w	1	1.01 (0.06, 16.08)	Not estimable	Not estimable
Duration > 52 w	3	0.75 (0.56, 1.01)	0	0.757
Class I (BMI 30–34.9 kg/m^2^)	1	0.75 (0.56, 0.99)	Not estimable	0.049
Class II (BMI 35–39.9 kg/m^2^)	3	1.40 (0.24, 8.06)	0	0.705
Angina pectoris				
Age ≤ 60 yr	2	0.50 (0.07, 3.55)	0	0.34
Age > 60 yr	1	0.77 (0.60, 0.98)	Not estimable	Not estimable
Duration ≤ 52 w	1	1.51 (0.06, 36.61)	Not estimable	Not estimable
Duration > 52 w	2	0.76 (0.60, 0.97)	0	0.35
Class I (BMI 30–34.9 kg/m^2^)	1	0.78 (0.61, 0.99)	Not estimable	0.039
Class II (BMI 35–39.9 kg/m^2^)	1	0.17 (0.01, 4.11)	Not estimable	0.274
Class III (BMI ≥ 40 kg/m^2^)	1	1.50 (0.06, 36.35)	Not estimable	0.803
Cardiac failure congestive				
Age ≤ 60 yr	1	0.22 (0.01, 5.47)	Not estimable	Not estimable
Age > 60 yr	2	0.79 (0.50, 1.27)	27.5	0.24
Duration ≤ 52 w	1	0.14 (0.01, 2.78)	Not estimable	Not estimable
Duration > 52 w	2	0.83 (0.51, 1.33)	0	0.416
Class I (BMI 30–34.9 kg/m^2^)	1	0.86 (0.53, 1.39)	Not estimable	0.542
Class II (BMI 35–39.9 kg/m^2^)	2	0.18 (0.02, 1.57)	0	0.121
Nephrolithiasis				
Age ≤ 60 yr	4	1.80 (0.37, 8.76)	0	0.987
Age > 60 yr	2	1.00 (0.58, 1.72)	0	0.565
Duration ≤ 52 w	1	0.50 (0.04, 5.54)	Not estimable	Not estimable
Duration > 52 w	5	1.11 (0.65, 1.88)	0	0.971
Class I (BMI 30–34.9 kg/m^2^)	2	1.05 (0.61, 1.83)	0	0.855
Class II (BMI 35–39.9 kg/m^2^)	4	1.16 (0.27, 5.00)	0	0.844
Acute kidney injury				
Age ≤ 60 yr	2	0.50 (0.07, 3.56)	0	0.341
Age > 60 yr	2	0.79 (0.59, 1.05)	66.7	0.083
Duration ≤ 52 w	1	5.05 (0.59, 42.99)	Not estimable	Not estimable
Duration > 52 w	3	0.74 (0.56, 0.99)	0	0.599
Class I (BMI 30–34.9 kg/m^2^)	2	0.76 (0.57, 1.01)	0	0.061
Class II (BMI 35–39.9 kg/m^2^)	1	4.98 (0.59, 42.35)	Not estimable	0.141
Class III (BMI ≥ 40 kg/m^2^)	1	0.17 (0.01, 4.12)	Not estimable	0.276
Ureterolithiasis				
Age ≤ 60 yr	2	0.84 (0.11, 6.34)	0	0.613
Age > 60 yr	1	2.00 (0.93, 4.26)	Not estimable	Not estimable
Duration > 52 w	3	1.80 (0.89, 3.65)	0	0.64
Class I (BMI 30–34.9 kg/m^2^)	3	1.80 (0.88, 3.67)	0	0.107

### Publication bias and sensitivity analysis

Funnel plots were constructed to assess potential publication bias for the outcomes of AF, acute myocardial infarction, and nephrolithiasis. The plots exhibited substantial symmetry (Figures S23–25), and the Egger test further supported these findings with *p* values of 0.707, 0.470, and 0.481, respectively, indicating no significant publication bias for these outcomes.

Sensitivity analyses were conducted to evaluate the stability of the results. For AF and acute myocardial infarction, Lincooff’s study was identified as having the most substantial influence on the overall effect sizes. In contrast, the findings for nephrolithiasis demonstrated greater robustness (Figures S26–28). These analyses highlight the varying degrees of influence of individual studies on the overall results.

### Assessment of study quality

The evaluation of study quality, as outlined in the risk of bias summary (Figure S29), revealed that the majority of the included studies [13–17, 19–22] were assessed as having a low risk of bias in critical areas, including random sequence generation, allocation concealment, and blinding. This reflects a generally satisfactory level of methodological rigor across the studies. Nevertheless, one study [18] was identified as having an unclear risk of bias, particularly in the blinding domain, which may introduce potential limitations to the findings.

## Discussion

This meta-analysis evaluated the effects of subcutaneous semaglutide on arrhythmic, major CV, and renal outcomes in patients with overweight or obesity. These findings demonstrate that semaglutide significantly reduces the risk of AF, sinus node dysfunction, acute myocardial infarction, and angina pectoris. However, its effects on other arrhythmic, CV, and renal outcomes were not statistically significant. First, these findings suggest that semaglutide may have a selective protective effect on specific arrhythmic outcomes, particularly AF and sinus node dysfunction, while its impact on other arrhythmias remains inconclusive. Further research is needed to clarify the mechanisms underlying these differential effects and to explore potential long-term benefits. Second, these results highlight the potential benefits of semaglutide in reducing acute myocardial infarction and angina pectoris, while its effects on other CV outcomes remain uncertain. These findings suggest that semaglutide may have a targeted effect on specific CV outcomes, warranting further investigation into its broader CV protective mechanisms. Third, these findings suggest that semaglutide does not significantly influence renal outcomes in patients with overweight or obesity. While it shows promise in CV and glycemic management, its role in renal protection remains unclear, highlighting the need for further research to explore its potential effects on kidney-related conditions. These results highlight the potential of semaglutide as a therapeutic option for managing CV risk in patients with overweight or obesity, while also underscoring the need for further research to clarify its broader impact.

The significant reduction in the risk of AF is one of the most notable findings of this study. AF is a common arrhythmia associated with obesity [23]. The mechanisms by which semaglutide reduces the risk of AF may be multifactorial. GLP-1 receptor activation has been shown to exert an anti-inflammatory action [24], which can reduce atrial inflammation—a key contributor to AF development. Furthermore, semaglutide may improve endothelial function and blood flow [25], which are crucial for maintaining normal electrical activity in the heart. The antiobesity effect of semaglutide lowers systemic fat mass and adipokine release, potentially reducing an important driver of atrial remodeling [26, 27]. Obesity is known to induce systemic inflammation and atrial remodeling, which are key drivers of AF. Semaglutide’s role in moderating inflammation caused by excess adipose tissue can inhibit the activation of pathways, such as the NLRP3 inflammasome [28], which contributes to atrial fibrosis and electrical disturbances. In addition, semaglutide may enhance glucose utilization and improve metabolic parameters, leading to reduced oxidative stress, another factor influencing atrial structural remodeling and arrhythmogenesis. Semaglutide, a GLP-1 receptor agonist, has been shown to reduce the expression of inflammatory markers such as C-reactive protein (CRP) and interleukin-6 (IL-6), potentially mitigating atrial remodeling and fibrosis [29, 30]. In addition, the ability of semaglutide to improve metabolic parameters [31], such as insulin sensitivity and lipid profiles, may further reduce the risk of AF. These mechanisms are supported by preclinical studies showing that GLP-1 receptor agonists reduce atrial fibrosis and oxidative stress in animal models of obesity. However, the lack of significant effects on other arrhythmias, such as AFL, ventricular tachycardia, and sinus bradycardia, suggests that the benefits of semaglutide may be specific to AF rather than a broad antiarrhythmic effect. This specificity may be due to semaglutide’s differential impact on adipose tissue and associated inflammatory processes, which interplay importantly in the pathophysiology of AF. Understanding these distinctions is critical for tailoring interventions aimed at other arrhythmias. This finding aligns with previous studies, such as the SUSTAIN-6 trial [32], which showed a neutral effect of semaglutide on overall arrhythmia risk but suggested a trend toward reduced AF incidence. Semaglutide demonstrated significant benefits in reducing the risk of acute myocardial infarction and angina pectoris. This can be attributed in part to its capacity to enhance endothelial function through nitric oxide (NO) release, improve lipid profiles, and reduce blood pressure. The resulting decrease in myocardial oxygen demand and ischemia can substantially decrease the risk of acute CV events. This underscores the importance of further investigating the comprehensive metabolic and hemodynamic changes facilitated by GLP-1 receptor agonists. These findings are consistent with the known CV protective effects of GLP-1 receptor agonists, which have been shown to improve endothelial function, reduce atherosclerotic plaque formation, and lower blood pressure. The weight loss and metabolic improvements associated with semaglutide likely contribute to these benefits, as obesity is a major risk factor for coronary artery disease and acute coronary syndrome. Specifically, semaglutide has been shown to reduce LDL cholesterol and triglycerides while increasing HDL cholesterol, all of which are important factors in reducing CV risk. However, the lack of significant effects on other CV outcomes, such as unstable angina, coronary artery disease, and heart failure, suggests that semaglutide’s CV benefits may be more pronounced in specific conditions rather than across the entire spectrum of CV diseases. This finding is consistent with findings from the PIONEER-6 trial [33], which showed a reduction in CV death and nonfatal myocardial infarction but no significant effect on heart failure hospitalization. Similarly, the STEP-HFpEF trial [19] demonstrated that semaglutide improved symptoms and physical limitations in patients with heart failure with preserved ejection fraction (HFpEF) but did not significantly reduce CV mortality. These findings suggest that semaglutide’s CV benefits may be context-dependent, with greater efficacy in certain patient populations or CV conditions.

The differential effects of semaglutide on various CV outcomes suggest its particular benefit in managing obesity-related comorbidities, such as acute myocardial infarction and AF. Semaglutide may effectively target pathophysiological mechanisms associated with obesity, including systemic inflammation and metabolic dysregulation, which drive conditions such as AF and myocardial ischemia. The absence of significant effects on other CV outcomes, such as heart failure or stable angina, indicates that its benefits may be context-specific and concentrated in scenarios where obesity and CV pathology converge. This highlights the necessity of personalized treatment strategies that consider the individual patient’s CV risk profile and prevalent conditions.

Additionally, while this meta-analysis focused primarily on arrhythmic and major CV outcomes, it is important to consider how these reductions may translate into improved patient-centered outcomes. Studies have demonstrated that reductions in arrhythmic events and major CV incidents often correlate with better quality of life and reduced hospitalizations. For patients with obesity, managing these CV risks not only enhances their physical health but could also improve their emotional well-being and overall mortality rates. Therefore, future research should aim to evaluate the broader impacts of semaglutide treatment on these crucial aspects of patient care.

In contrast to its CV benefits, semaglutide did not significantly affect renal outcomes, including nephrolithiasis, acute kidney injury, or chronic kidney disease. This is somewhat surprising, given the known association between obesity and renal complications, as well as the potential renoprotective effects of GLP-1 receptor agonists. However, our findings indicate that renoprotective benefits may not extend to nondiabetic patients with obesity or overweight. The lack of significant effects on renal outcomes in this population may be due to the relatively short duration of follow-up in some studies or the inclusion of patients with less severe renal impairment. Further research is needed to explore whether semaglutide has renoprotective effects in specific subgroups, such as those with pre-existing kidney disease or more severe obesity.

Semaglutide has shown promising effects in managing CV outcomes compared to other GLP-1 receptor agonists, such as liraglutide and dulaglutide. While liraglutide has demonstrated CV benefits in high-risk patients through trials such as LEADER [34], its effect sizes often appear less pronounced than those reported for semaglutide. For example, the SUSTAIN-6 trial indicated that semaglutide reduced the risk of major adverse CV events significantly more than liraglutide [35]. Moreover, the enhanced potency and receptor selectivity of semaglutide, which allows for once-weekly dosing, contribute to better adherence and weight loss outcomes, potentially offering superior overall benefits. However, further head-to-head studies are necessary to establish the comparative effectiveness and safety profiles of each agent, particularly concerning various CV conditions and patient populations.

Subgroup analyses revealed that the benefits of semaglutide were more pronounced in patients over 60 years of age and those with treatment durations exceeding 52 weeks. For example, the risk of acute myocardial infarction was significantly lower in older patients and those with longer treatment durations. Similarly, the risk of angina pectoris was significantly lower in older patients and those with longer treatment durations. These findings suggest that semaglutide may be particularly beneficial for older patients, who are at higher risk for CV events, and that longer treatment durations may be necessary to achieve significant benefits. This finding has important implications for clinical practice, as it highlights the importance of considering patient age and treatment duration when prescribing semaglutide. However, it is important to acknowledge potential confounding factors, such as the presence of comorbidities that may differ significantly between age groups. Additionally, while these subgroup results are promising, caution should be exercised in generalizing these findings to broader populations. The specific characteristics of the study participants, such as socioeconomic status, concomitant medications, and the overall health status of patients with obesity, may not fully represent the diverse demographics found in real-world settings. Age plays a critical role in clinical decision-making for prescribing semaglutide, particularly given the increasing prevalence of obesity and CV diseases in older populations. Our subgroup analysis indicates that elderly patients demonstrated pronounced benefits from semaglutide treatment in reducing the risk of acute myocardial infarction and angina pectoris. This highlights a fundamental consideration in treatment strategies; as older adults are at heightened risk for CV events due to age-related physiological changes and comorbidities, tailoring treatment plans that prioritize the specific benefits of semaglutide in this demographic may enhance outcomes. Hence, clinicians should factor in age alongside obesity classification when evaluating potential treatment pathways, ensuring that the therapeutic approach aligns with the patient’s risk profile and health status.

Additionally, subgroup analyses based on obesity levels indicated that semaglutide was particularly effective among patients classified with Class I obesity (BMI 30–34.9 kg/m^2^), where a significant reduction in the risk of acute myocardial infarction was observed. However, the effectiveness appeared to diminish in patients with Class II and Class III obesity, suggesting that the therapeutic response to semaglutide may vary across different obesity levels. This underscores the need for a personalized approach to treatment, taking into account both age and BMI when determining the potential benefits of semaglutide for patients. The low heterogeneity (*I*^2^ = 0) observed in most analyses indicates a high degree of consistency across studies, which strengthens the reliability of our findings. However, some outcomes, such as acute kidney injury and renal failure, showed moderate to high heterogeneity, which may be due to differences in study populations, definitions of outcomes, or follow-up durations. The absence of significant publication bias, as indicated by symmetrical funnel plots and nonsignificant Egger’s test results, further supports the robustness of our findings. Nevertheless, the limited number of studies for some outcomes, particularly renal outcomes, suggests that caution is warranted when interpreting these results. Notably, all studies included in our analysis administered a consistent dosage of 2.4 mg semaglutide. Unfortunately, original studies did not provide comprehensive data on comorbidities such as hypertension or diabetes, limiting our ability to analyze their effects on treatment response.

Despite the positive findings associated with semaglutide, it is crucial to acknowledge the need for a balanced assessment of its safety profile. Common adverse events reported in clinical trials include gastrointestinal disturbances such as nausea, vomiting, and diarrhea, which may affect adherence to treatment [36]. Although these side effects are generally mild to moderate and tend to diminish over time, clinicians should be aware of their potential impact on patient adherence and quality of life. Ongoing monitoring of adverse events and patient feedback will be important for the long-term management of patients treated with semaglutide.

This study builds on previous work to further understand the role of semaglutide in several CV and metabolic domains. Previous meta-analyses have focused on weight changes and gastrointestinal side effects of semaglutide in patients with obesity or overweight. For example, Kommu et al. [37] reported that, compared with placebo, semaglutide reduced changes in body weight (MD −11.49%, −13.12 to −9.86), absolute body weight (MD −11.74 kg, 95% CI −13.53 to −19.94), and waist circumference (MD −9.06 cm, −10.33 to −7.79). Furthermore, Qin et al. [38] found that semaglutide increased the risk of gastrointestinal side effects (RR 1.49, 95% CI 1.38–1.60). Although some studies have also evaluated AF outcomes, such as those of Saglietto et al. [39] and Zhang et al. [40], they confounded patients with type 2 diabetes, which may not be applicable to analyzing patients who are merely obese or overweight without diabetes. Furthermore, our study contrasts with the findings of Patoulias et al. [41], who reported that semaglutide did not reduce AF compared with placebo (RR 0.71, 95% CI 0.21–2.34). Based on the singularity of these studies, our study therefore expands the underexplored area: the specific effects of semaglutide on various outcomes, such as AFL, atrial tachycardia, and angina pectoris. Moreover, major CV and renal outcomes have been of greater interest. The study by Singh et al. [41] found that GLP-1 receptor agonists significantly reduce the risk of myocardial infarction in patients with overweight or obesity (OR 0.72, 95% CI 0.61–0.85), whereas our study revealed that semaglutide does not increase the risk of myocardial infarction (RR 0. 54, 95% CI 0.06–4.84). The study by Hu et al. [42] found that semaglutide significantly reduced the risk of new or worsening nephropathy (HR 0.64, 95% CI 0.46–0.88) and macroalbuminuria (HR 0.54, 95% CI 0.37–0.77) in patients with overweight or obesity (HR 0.89, 95% CI 0.82–0.98), and that conclusion is based on the SUSTAIN-6 study, for patients with type 2 diabetes. Therefore, we collected a number of renal endpoints to evaluate semiglutide. Overall, this study not only confirms the CV benefits of semaglutide but also emphasizes its potential to reduce the risk of arrhythmias. These findings fill an important gap in the literature and pave the way for further research.

This meta-analysis offers several notable strengths that enhance its contribution to the field. First, it provides a comprehensive evaluation of subcutaneous semaglutide effects on a wide range of outcomes, including arrhythmic, major CV, and renal outcomes, specifically in patients with overweight or obesity. This focus addresses a critical gap in the literature, as previous research has primarily centered on patients with type 2 diabetes. Second, the study adhered to the PRISMA guidelines and employed a rigorous methodology, including a systematic search of multiple databases, strict inclusion and exclusion criteria, and the use of a random-effects model to account for heterogeneity. The risk of bias was assessed via the Cochrane Collaboration tool, ensuring the quality and reliability of the included studies. Third, the inclusion of subgroup analyses based on age, treatment duration and obesity level provides valuable insights into the differential effects of semaglutide, highlighting that older patients and those with longer treatment durations may derive greater benefits. This finding has important implications for personalized treatment strategies. Additionally, most analyses demonstrated low heterogeneity, indicating a high degree of consistency across studies, and the absence of significant publication bias further strengthened the reliability of the findings. Finally, the study results have direct clinical relevance, particularly in supporting the use of semaglutide for managing CV risks in patients with overweight or obesity, as evidenced by the significant reduction in the risk of AF, acute myocardial infarction, and angina pectoris. However, this meta-analysis has several limitations. First, the number of studies included for some outcomes, particularly renal outcomes, was relatively small, which may limit the generalizability of the findings. Second, the follow-up durations in some studies were relatively short, which may not capture the long-term effects of semaglutide on CV and renal outcomes. Third, the inclusion criteria for obesity and overweight varied across studies, which may introduce heterogeneity. Finally, the meta-analysis was limited to subcutaneous semaglutide, and the results may not be generalizable to oral formulations.

Several key research directions should be prioritized to build on the findings of this study and address existing gaps in knowledge. First, future studies should investigate the long-term effects of semaglutide on arrhythmic, CV, and renal outcomes, as the current evidence is limited by relatively short follow-up durations in most trials. In addition, mechanistic studies are essential to elucidate the molecular and physiological pathways through which semaglutide exerts its effects, particularly its anti-inflammatory and antifibrotic effects, which may play critical roles in mitigating AF and other CV diseases. Second, the research should focus on specific patient subgroups, such as those with pre-existing kidney disease or severe obesity, to clarify semaglutide’s renoprotective potential, given the lack of significant effects on renal outcomes in this study. Third, comparative studies with other GLP-1 receptor agonists, such as liraglutide and dulaglutide, are needed to determine whether the observed benefits are class effects or specific to semaglutide. Finally, real-world evidence should be incorporated to evaluate the effectiveness and safety of semaglutide in diverse patient populations and settings, as RCTs may not fully reflect clinical practice. By addressing these gaps, future research can further clarify the role of semaglutide in managing obesity-related complications and optimize its use in clinical practice.

## Conclusion

This systematic review and meta-analysis demonstrated that semaglutide significantly reduces the risk of AF, sinus node dysfunction, acute myocardial infarction, and angina pectoris in patients with overweight or obesity. These findings highlight its cardioprotective potential beyond glycemic control. While no significant effects were observed for other arrhythmic or renal outcomes, the overall evidence supports semaglutide as a valuable therapeutic option for managing CV risk in patients with overweight or obesity. Based on our findings, we recommend that healthcare providers consider semaglutide as a therapeutic option for patients with overweight or obesity, especially in older adults and those at high risk for CV events. Future studies should prioritize investigating the long-term effects of semaglutide on CV and renal outcomes, given the current limitations of follow-up durations in clinical trials. Furthermore, it is imperative that healthcare providers consider the potential benefits of improved quality of life and reduced hospitalizations for patients undergoing treatment with semaglutide, alongside an awareness of its safety profile and side effects.

## Supplementary Information

Supplementary material 1.

## Data Availability

The datasets used and/or analyzed during the current study are available from the corresponding author upon reasonable request.
