# Supplementary material for: Effect of semaglutide on arrhythmic, major cardiovascular, and renal outcomes in patients with overweight or obesity: a systematic review and meta-analysis
Source: Eur J Med Res. 2025 Sep 2;30:835. doi: 10.1186/s40001-025-03124-y (PMC12403603; doi:10.1186/s40001-025-03124-y)
Supplement: Supplementary file 1 — Supplementary material 1. [file 40001_2025_3124_MOESM1_ESM.docx]

**Supplementary Information**

Table of Contents

[Supplement 1: Search strategies 2](#_Toc190442014)

[Supplement 2: Forest plot of arrhythmic outcomes 2](#_Toc190442015)

[Supplement 3: Forest plot of major cardiovascular outcomes 3](#_Toc190442016)

[Supplement 4: Forest plot of renal outcomes 3](#_Toc190442017)

[Supplement 5: Funnel plot 4](#_Toc190442018)

[Supplement 6: Sensitivity analysis 4](#_Toc190442019)

[Supplement 7: The risk of bias summary 5](#_Toc190442020)

[Supplement 8: PRISMA checklist 6](#_Toc190442021)

# Supplement 1: Search strategies

| **Database** | **Search strategy** | **Results** |
| --- | --- | --- |
| **Pubmed** | **((("Semaglutide"[Supplementary Concept] OR Ozempic[Title/Abstract] OR rybelsus[Title/Abstract] OR Wegovy[Title/Abstract])) AND (("Overweight"[Mesh] OR "Obesity"[Mesh]))) AND ((randomized controlled trial[Publication Type] OR randomized[Title/Abstract] OR placebo[Title/Abstract]))** | **120** |
| **EMBASE** | **('Semaglutide'/exp OR ‘Ozempic’:ti,ab OR ‘rybelsus’:ti,ab OR ‘Wegovy’:ti,ab) AND ('Overweight'/exp OR 'Obesity'/exp) AND** **(‘random’:ti,ab OR ‘placebo’:ti,ab OR ‘double-blind’:ti,ab)** | **430** |
| **Cochrane** | **(Semaglutide:ti,ab,kw OR Ozempic:ti,ab,kw OR rybelsus:ti,ab,kw OR Wegovy:ti,ab,kw) AND (Overweight:ti,ab,kw OR Obesity:ti,ab,kw)** | **519** |

*** All searches were carried out on January 20, 2025.**

# Supplement 2: Forest plot of arrhythmic outcomes


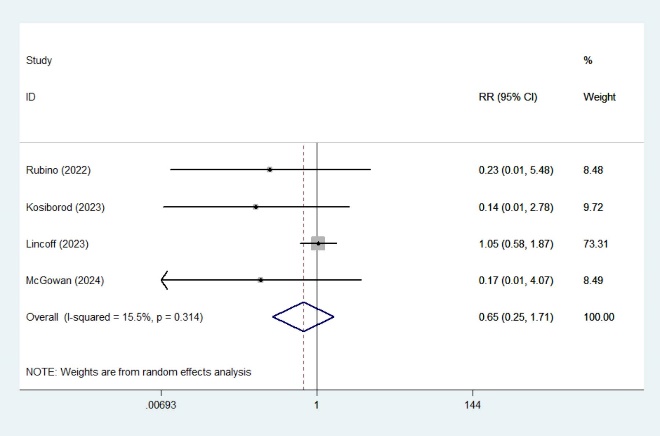


**Figure S1.** **Forest plot of atrial flutter (AFL).**


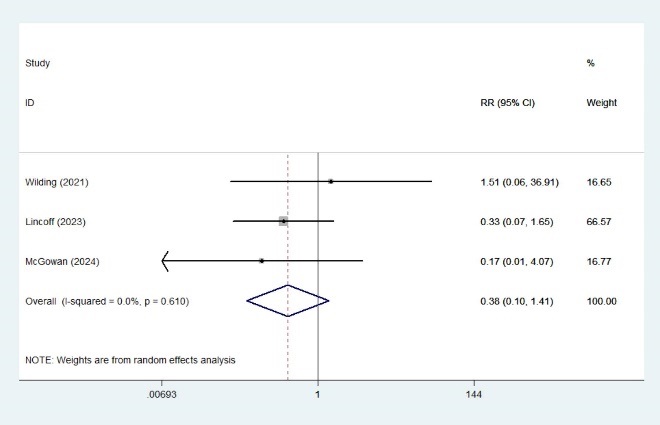


**Figure S2.** **Forest plot of atrial tachycardia.**


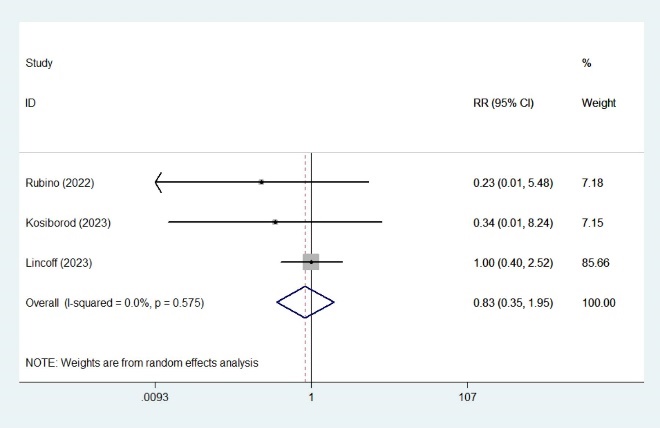


**Figure S3.** **Forest plot of bradyarrhythmia.**


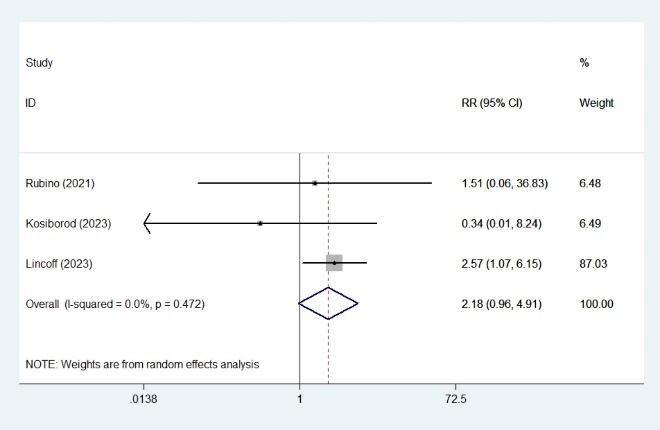


**Figure S4.** **Forest plot of supraventricular tachycardia.**


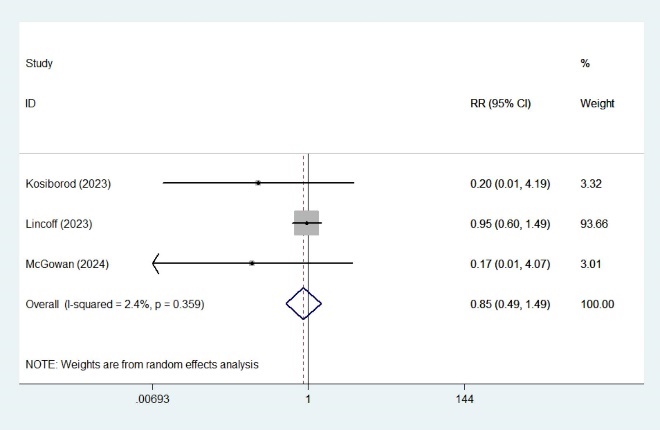


**Figure S5.** **Forest plot of ventricular tachycardia.**


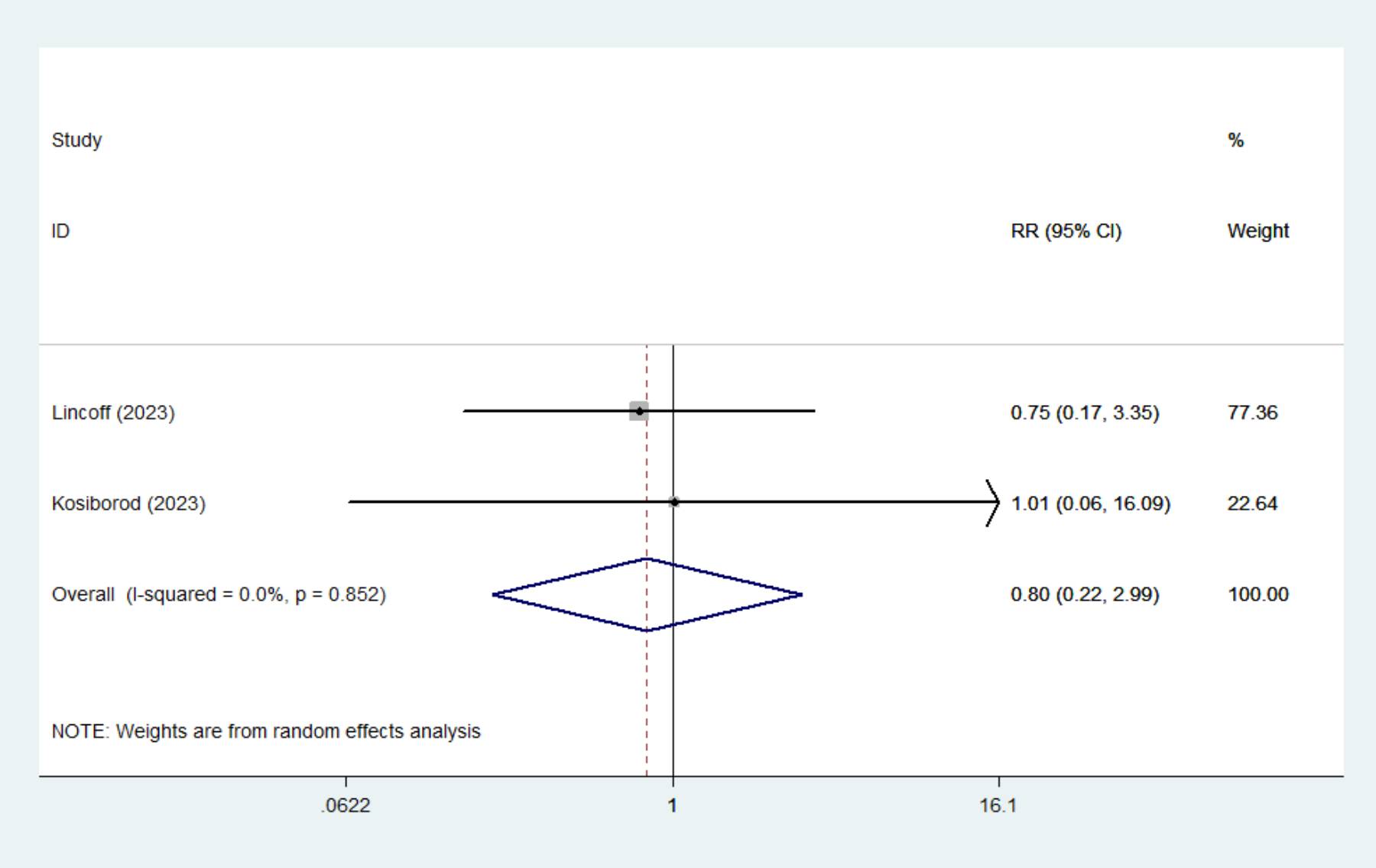


**Figure S6.** **Forest plot of sinus bradycardia.**


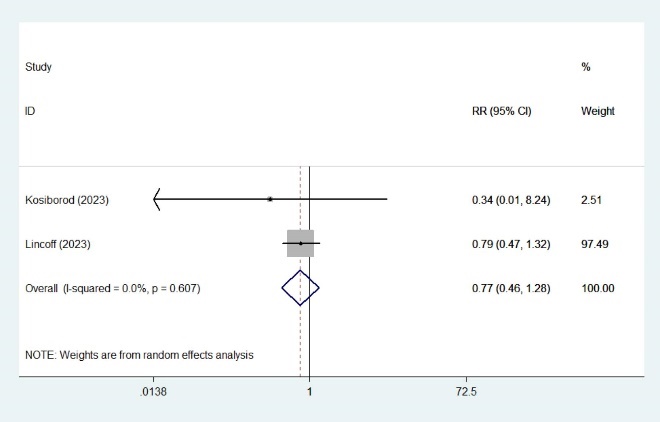


**Figure S7.** **Forest plot of cardiac arrest.**

# Supplement 3: Forest plot of major cardiovascular outcomes


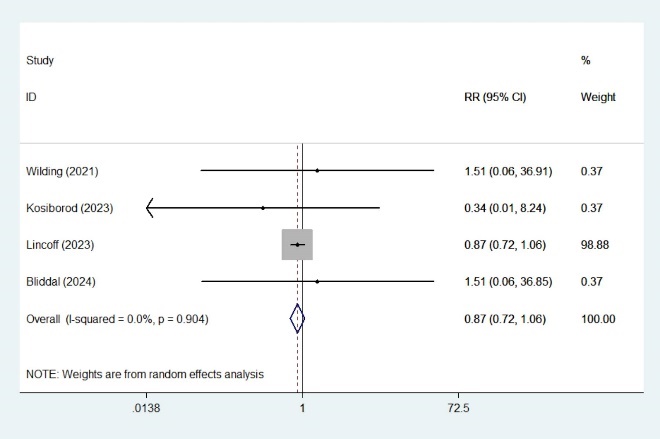


**Figure S8.** **Forest plot of angina unstable.**


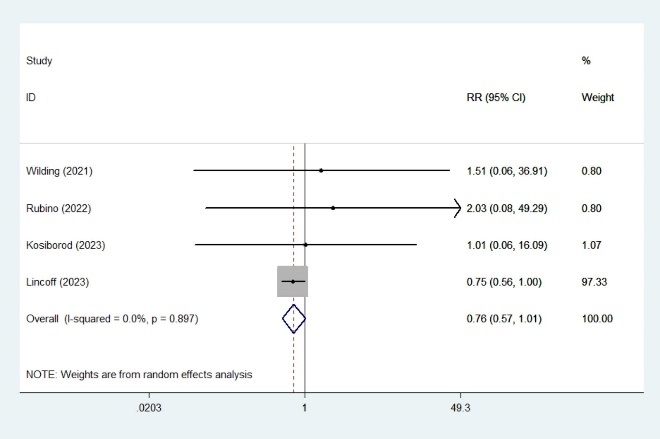


**Figure S9.** **Forest plot of coronary artery disease.**


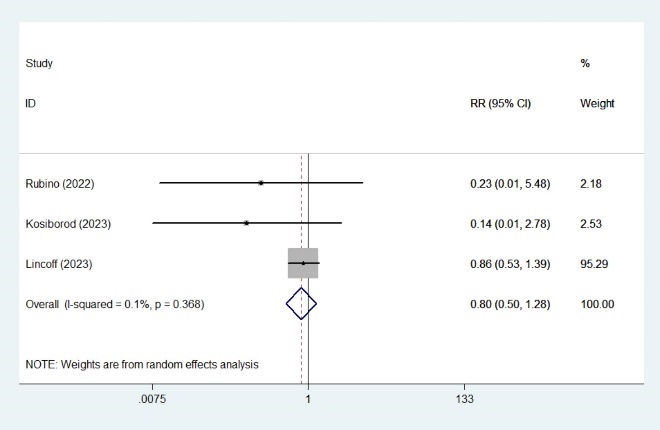


**Figure S10.** **Forest plot of cardiac failure congestive.**


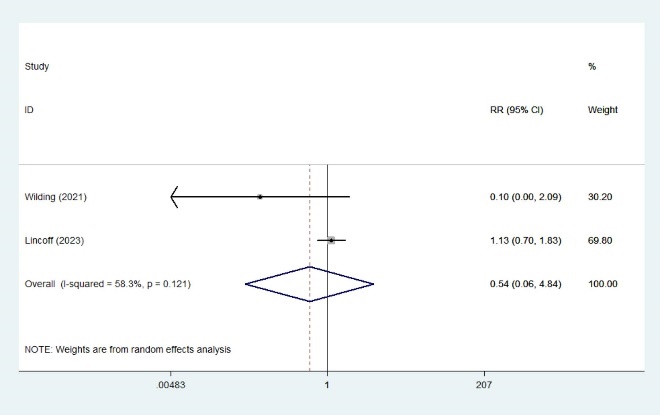


**Figure S11.** **Forest plot of myocardial infarction.**


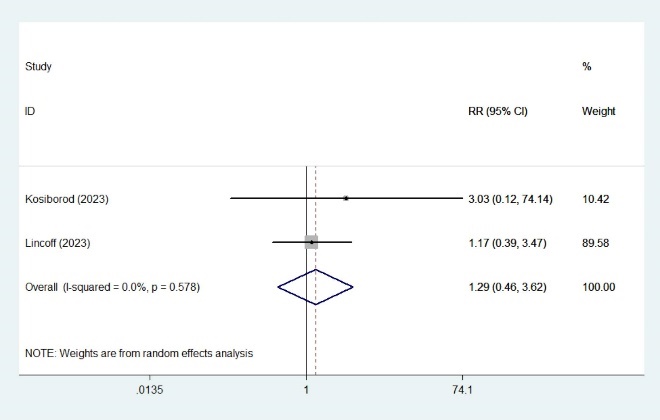


**Figure S12.** **Forest plot of aortic valve stenosis.**


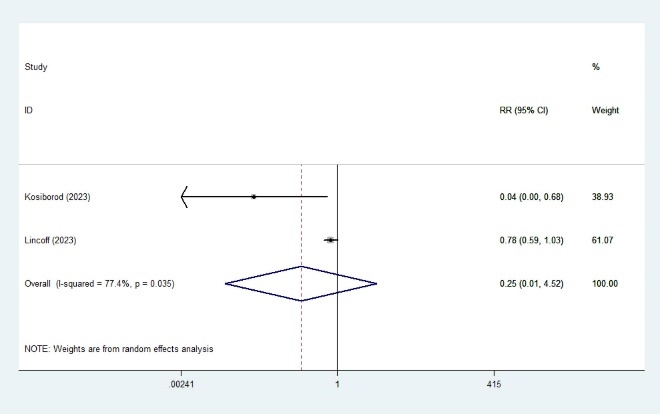


**Figure S13.** **Forest plot of cardiac failure.**


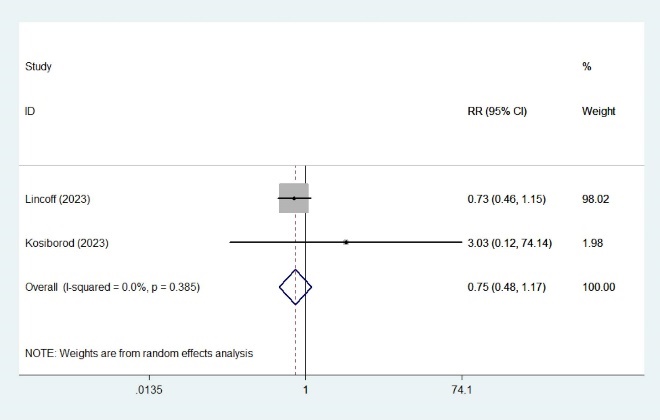


**Figure S14.** **Forest plot of cardiac failure acute.**


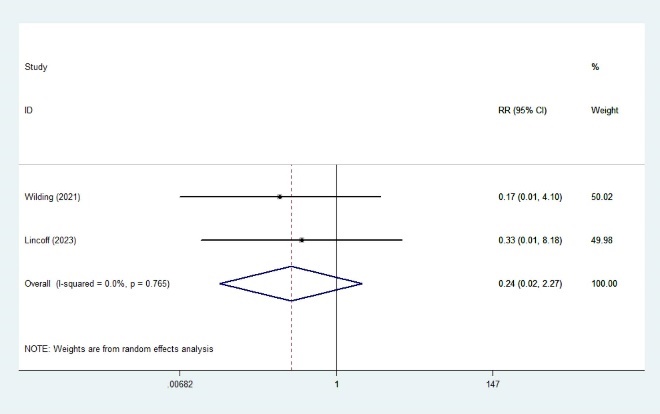


**Figure S15.** **Forest plot of myocarditis.**


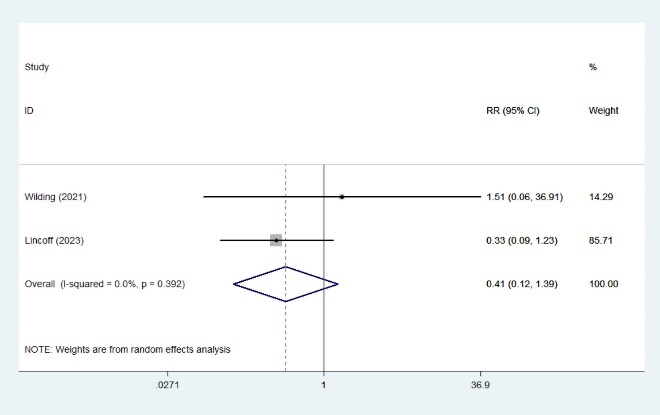


**Figure S16.** **Forest plot of palpitations.**

# Supplement 4: Forest plot of renal outcomes


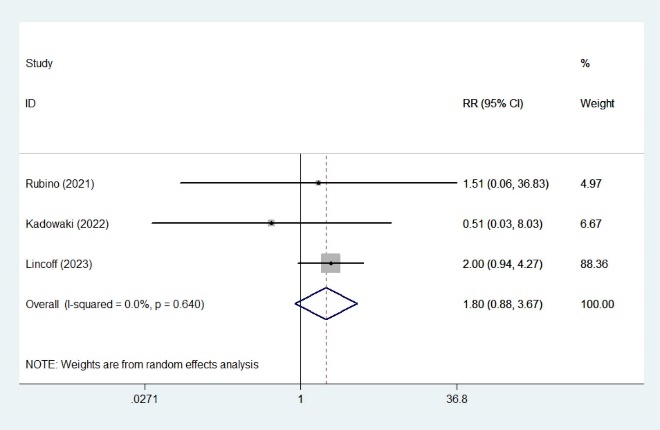


**Figure S17.** **Forest plot of ureterolithiasis.**


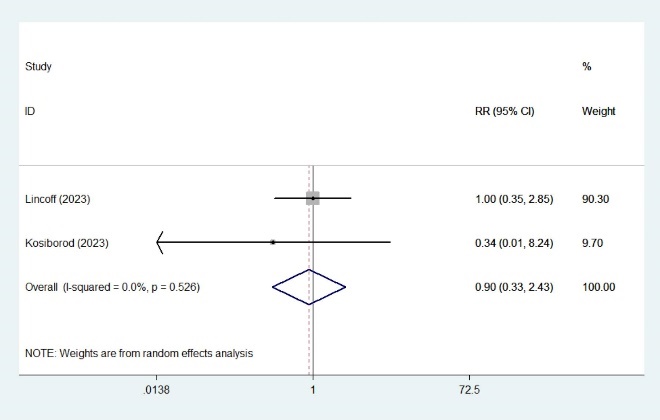


**Figure S18.** **Forest plot of chronic kidney disease.**


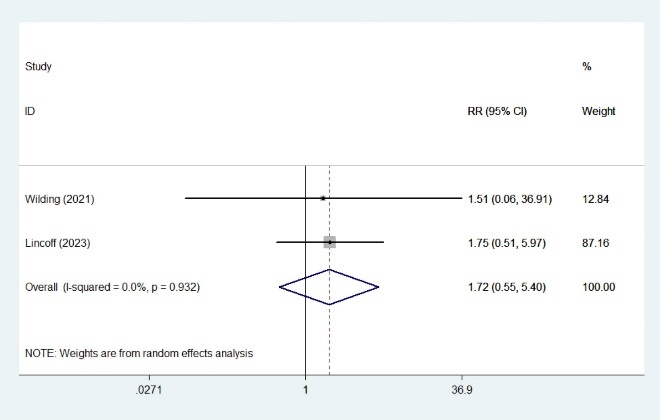


**Figure S19.** **Forest plot of calculus urinary.**


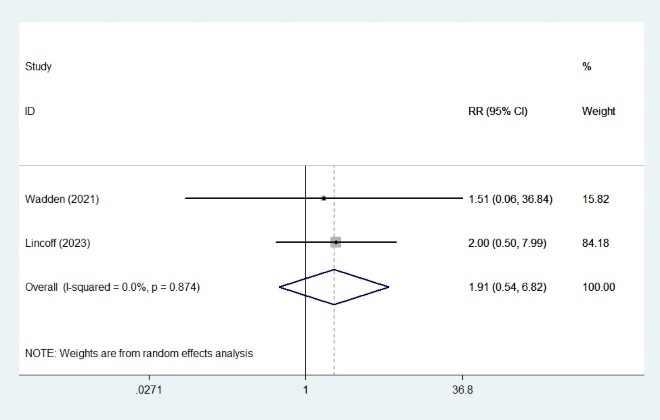


**Figure S20.** **Forest plot of hydronephrosis.**


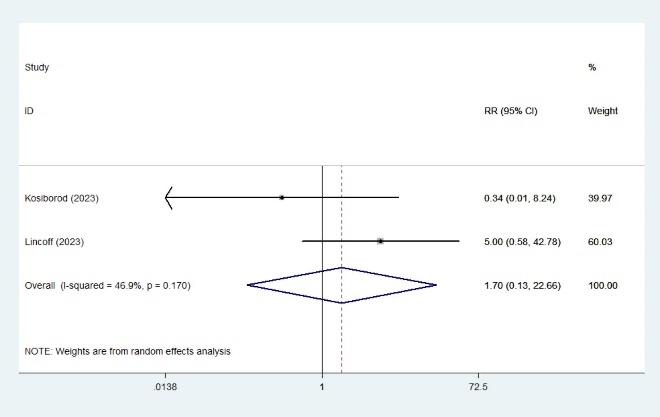


**Figure S21.** **Forest plot of renal colic.**


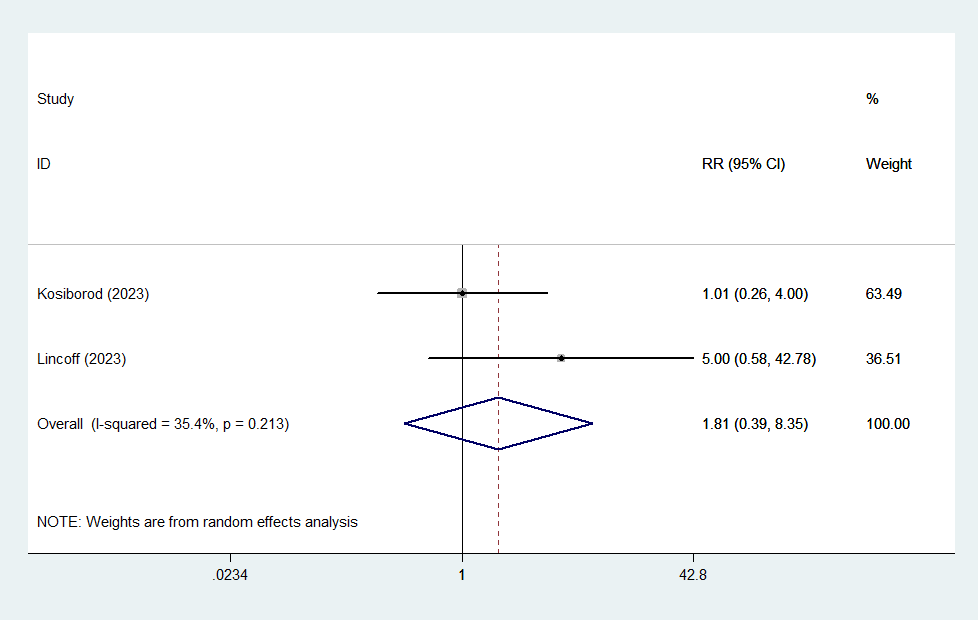


**Figure S22.** **Forest plot of renal failure.**

# Supplement 5: Funnel plot


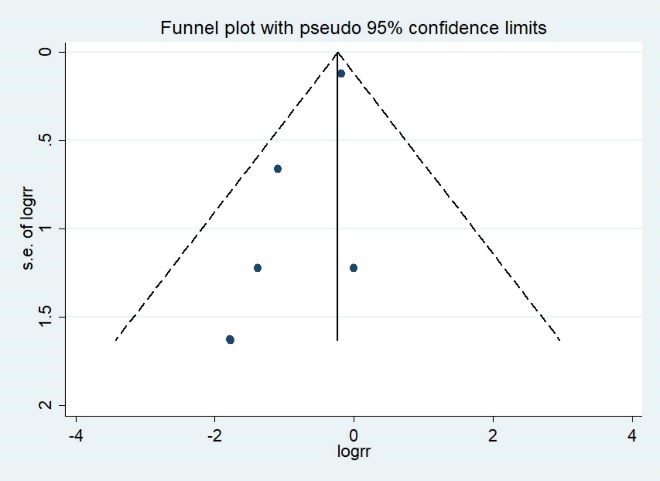


**Figure S23.** **Forest plot of atrial fibrillation.**


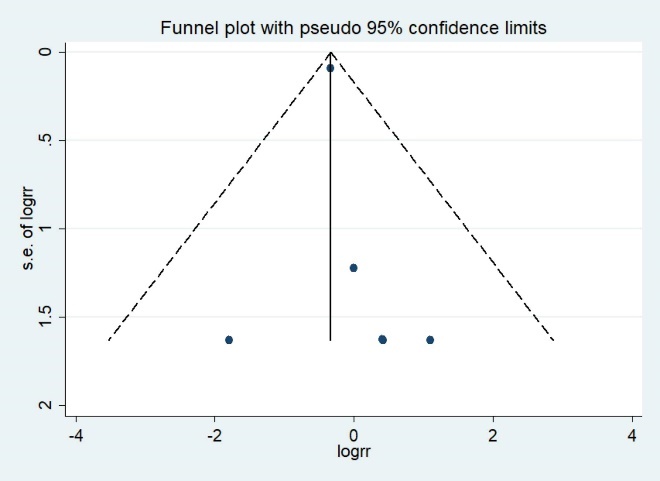


**Figure S24.** **Forest plot of acute myocardial infarction.**


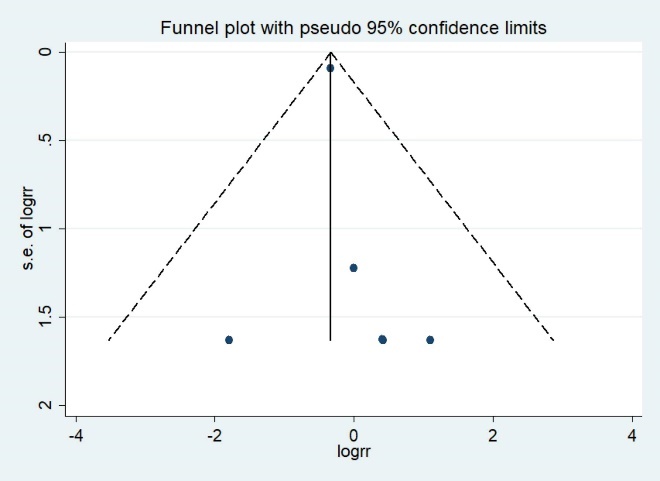


**Figure S25.** **Forest plot of nephrolithiasis.**

# Supplement 6: Sensitivity analysis


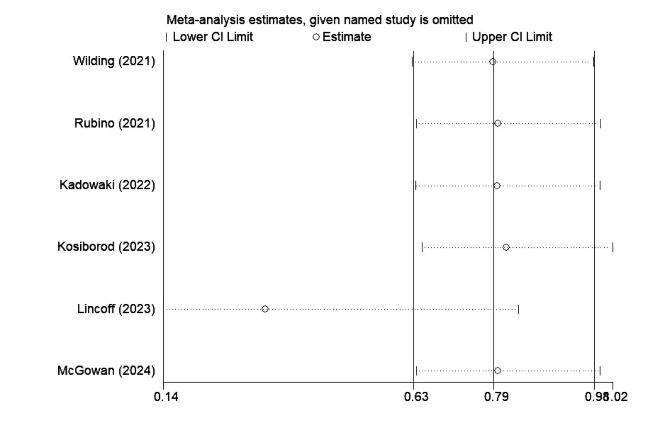


**Figure S26.** **Sensitivity analysis of atrial fibrillation.**


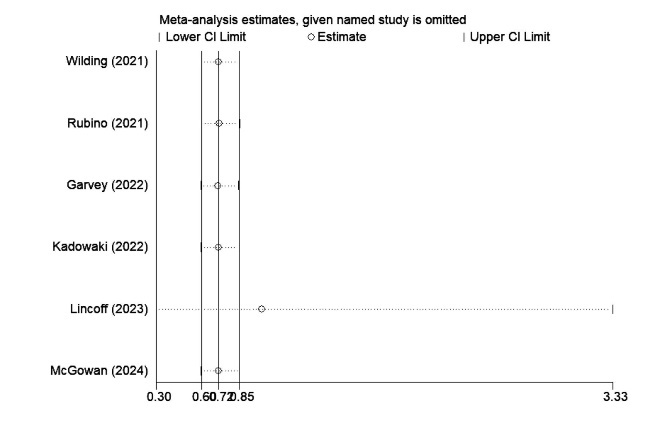


**Figure S27.** **Sensitivity analysis of acute myocardial infarction.**


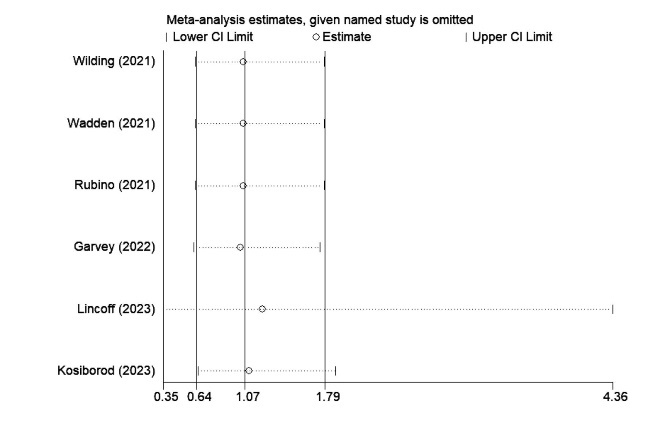


**Figure S28.** **Sensitivity analysis of nephrolithiasis.**

# Supplement 7: The risk of bias summary


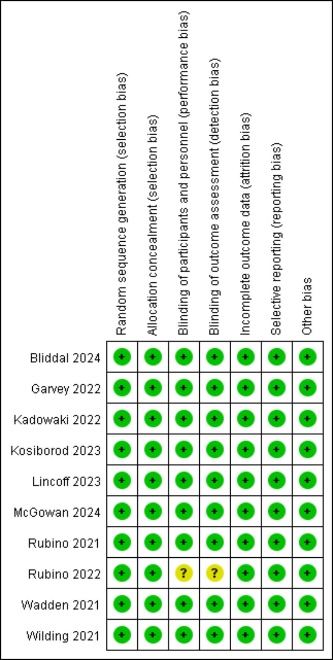


**Figure S29.** **The risk of bias summary.**

# Supplement 8: PRISMA checklist

| **Section and Topic** | **No.** | **Checklist item** | **Location where item is reported** |
| --- | --- | --- | --- |
| **TITLE** | | |  |
| Title | 1 | Identify the report as a systematic review. | Page 01 |
| **ABSTRACT** | | |  |
| Abstract | 2 | See the PRISMA 2020 for Abstracts checklist. | Page 02 |
| **INTRODUCTION** | | |  |
| Rationale | 3 | Describe the rationale for the review in the context of existing knowledge. | Page 02 |
| Objectives | 4 | Provide an explicit statement of the objective(s) or question(s) the review addresses. | Page 03 |
| **METHODS** | | |  |
| Eligibility criteria | 5 | Specify the inclusion and exclusion criteria for the review and how studies were grouped for the syntheses. | Page 03 |
| Information sources | 6 | Specify all databases, registers, websites, organisations, reference lists and other sources searched or consulted to identify studies. Specify the date when each source was last searched or consulted. | Page 03 |
| Search strategy | 7 | Present the full search strategies for all databases, registers and websites, including any filters and limits used. | Page 03 |
| Selection process | 8 | Specify the methods used to decide whether a study met the inclusion criteria of the review, including how many reviewers screened each record and each report retrieved, whether they worked independently, and if applicable, details of automation tools used in the process. | Page 03 |
| Data collection process | 9 | Specify the methods used to collect data from reports, including how many reviewers collected data from each report, whether they worked independently, any processes for obtaining or confirming data from study investigators, and if applicable, details of automation tools used in the process. | Page 04 |
| Data items | 10a | List and define all outcomes for which data were sought. Specify whether all results that were compatible with each outcome domain in each study were sought (e.g. for all measures, time points, analyses), and if not, the methods used to decide which results to collect. | Page 04 |
|  | 10b | List and define all other variables for which data were sought (e.g. participant and intervention characteristics, funding sources). Describe any assumptions made about any missing or unclear information. | Page 04 |
| Study risk of bias assessment | 11 | Specify the methods used to assess risk of bias in the included studies, including details of the tool(s) used, how many reviewers assessed each study and whether they worked independently, and if applicable, details of automation tools used in the process. | Page 04 |
| Effect measures | 12 | Specify for each outcome the effect measure(s) (e.g. risk ratio, mean difference) used in the synthesis or presentation of results. | Page 04 |
| Synthesis methods | 13a | Describe the processes used to decide which studies were eligible for each synthesis (e.g. tabulating the study intervention characteristics and comparing against the planned groups for each synthesis (item #5)). | Page 04 |
|  | 13b | Describe any methods required to prepare the data for presentation or synthesis, such as handling of missing summary statistics, or data conversions. | Page 04 |
|  | 13c | Describe any methods used to tabulate or visually display results of individual studies and syntheses. | Page 04 |
|  | 13d | Describe any methods used to synthesize results and provide a rationale for the choice(s). If meta-analysis was performed, describe the model(s), method(s) to identify the presence and extent of statistical heterogeneity, and software package(s) used. | Page 04 |
|  | 13e | Describe any methods used to explore possible causes of heterogeneity among study results (e.g. subgroup analysis, meta-regression). | Page 04 |
|  | 13f | Describe any sensitivity analyses conducted to assess robustness of the synthesized results. | Page 04 |
| Reporting bias assessment | 14 | Describe any methods used to assess risk of bias due to missing results in a synthesis (arising from reporting biases). | Page 04 |
| Certainty assessment | 15 | Describe any methods used to assess certainty (or confidence) in the body of evidence for an outcome. | Page 04 |
| **RESULTS** | | |  |
| Study selection | 16a | Describe the results of the search and selection process, from the number of records identified in the search to the number of studies included in the review, ideally using a flow diagram. | Page 04 |
|  | 16b | Cite studies that might appear to meet the inclusion criteria, but which were excluded, and explain why they were excluded. | Page 04 |
| Study characteristics | 17 | Cite each included study and present its characteristics. | Page 05 |
| Risk of bias in studies | 18 | Present assessments of risk of bias for each included study. | Page 06 |
| Results of individual studies | 19 | For all outcomes, present, for each study: (a) summary statistics for each group (where appropriate) and (b) an effect estimate and its precision (e.g. confidence/credible interval), ideally using structured tables or plots. | Page 05 |
| Results of syntheses | 20a | For each synthesis, briefly summarise the characteristics and risk of bias among contributing studies. | Page 05 |
|  | 20b | Present results of all statistical syntheses conducted. If meta-analysis was done, present for each the summary estimate and its precision (e.g. confidence/credible interval) and measures of statistical heterogeneity. If comparing groups, describe the direction of the effect. | Page 05 |
|  | 20c | Present results of all investigations of possible causes of heterogeneity among study results. | Page 06 |
|  | 20d | Present results of all sensitivity analyses conducted to assess the robustness of the synthesized results. | Page 06 |
| Reporting biases | 21 | Present assessments of risk of bias due to missing results (arising from reporting biases) for each synthesis assessed. | Page 06 |
| Certainty of evidence | 22 | Present assessments of certainty (or confidence) in the body of evidence for each outcome assessed. | Page 06 |
| **DISCUSSION** | | |  |
| Discussion | 23a | Provide a general interpretation of the results in the context of other evidence. | Page 07-10 |
|  | 23b | Discuss any limitations of the evidence included in the review. | Page 07-10 |
|  | 23c | Discuss any limitations of the review processes used. | Page 07-10 |
|  | 23d | Discuss implications of the results for practice, policy, and future research. | Page 10 |
| **OTHER INFORMATION** | | |  |
| Registration and protocol | 24a | Provide registration information for the review, including register name and registration number, or state that the review was not registered. | Page 03 |
|  | 24b | Indicate where the review protocol can be accessed, or state that a protocol was not prepared. | Page 03 |
|  | 24c | Describe and explain any amendments to information provided at registration or in the protocol. | Page 03 |
| Support | 25 | Describe sources of financial or non-financial support for the review, and the role of the funders or sponsors in the review. | Page 11 |
| Competing interests | 26 | Declare any competing interests of review authors. | Page 11 |
| Availability of data, code and other materials | 27 | Report which of the following are publicly available and where they can be found: template data collection forms; data extracted from included studies; data used for all analyses; analytic code; any other materials used in the review. | Page 11 |
